# Single-cell genomic and transcriptomic landscapes of primary and metastatic colorectal cancer tumors

**DOI:** 10.1186/s13073-022-01093-z

**Published:** 2022-08-16

**Authors:** Rui Wang, Jingyun Li, Xin Zhou, Yunuo Mao, Wendong Wang, Shuai Gao, Wei Wang, Yuan Gao, Kexuan Chen, Shuntai Yu, Xinglong Wu, Lu Wen, Hao Ge, Wei Fu, Fuchou Tang

**Affiliations:** 1 Biomedical Pioneering Innovation Center, Department of General Surgery, School of Life Sciences, Third Hospital, Peking University, Beijing, 100871 People’s Republic of China; 2 Beijing Advanced Innovation Center for Genomics & Key Laboratory of Assisted Reproduction, Ministry of Education, Beijing, 100871 People’s Republic of China; 3 Academy for Advanced Interdisciplinary Studies, Peking University, Beijing, 100871 People’s Republic of China; 4 Peking-Tsinghua Center for Life Sciences, Peking University, Beijing, 100871 People’s Republic of China; 5 Peking University Third Hospital Cancer Center, Beijing, 100193 China; 6 College of Animal Science and Technology, China Agricultural University, Beijing, 100193 China; 7 Beijing International Center for Mathematical Research, Peking University, Beijing, 100871 People’s Republic of China

**Keywords:** Metastatic colorectal cancer, Single-cell transcriptome profiling, Lineage tracing, Genotype-phenotype relationship, Mitochondrial mutations, Patient-derived organoids, PPAR signaling pathway

## Abstract

**Background:**

Colorectal cancer (CRC) ranks as the second-leading cause of cancer-related death worldwide with metastases being the main cause of cancer-related death. Here, we investigated the genomic and transcriptomic alterations in matching adjacent normal tissues, primary tumors, and metastatic tumors of CRC patients.

**Methods:**

We performed whole genome sequencing (WGS), multi-region whole exome sequencing (WES), simultaneous single-cell RNA-Seq, and single-cell targeted cDNA Sanger sequencing on matching adjacent normal tissues, primary tumors, and metastatic tumors from 12 metastatic colorectal cancer patients (*n*=84 for genomes, *n*=81 for exomes, *n*=9120 for single cells). Patient-derived tumor organoids were used to estimate the anti-tumor effects of a PPAR inhibitor, and self-renewal and differentiation ability of stem cell-like tumor cells.

**Results:**

We found that the PPAR signaling pathway was prevalently and aberrantly activated in CRC tumors. Blocking of PPAR pathway both suppressed the growth and promoted the apoptosis of CRC organoids in vitro, indicating that aberrant activation of the PPAR signaling pathway plays a critical role in CRC tumorigenesis. Using matched samples from the same patient, distinct origins of the metastasized tumors between lymph node and liver were revealed, which was further verified by both copy number variation and mitochondrial mutation profiles at single-cell resolution. By combining single-cell RNA-Seq and single-cell point mutation identification by targeted cDNA Sanger sequencing, we revealed important phenotypic differences between cancer cells with and without critical point mutations (*KRAS* and *TP53*) in the same patient in vivo at single-cell resolution.

**Conclusions:**

Our data provides deep insights into how driver mutations interfere with the transcriptomic state of cancer cells in vivo at a single-cell resolution. Our findings offer novel knowledge on metastatic mechanisms as well as potential markers and therapeutic targets for CRC diagnosis and therapy. The high-precision single-cell RNA-seq dataset of matched adjacent normal tissues, primary tumors, and metastases from CRCs may serve as a rich resource for further studies.

**Supplementary Information:**

The online version contains supplementary material available at 10.1186/s13073-022-01093-z.

## Background

Colorectal cancer (CRC) is the second-leading cause of cancer-related death worldwide [1, 2]. In CRC patients, metastases are the main cause of cancer-related death and liver is one of the common metastatic sites (accounting for 70% of all CRC patients with metastatic cancer) [3, 4]. The 5-year overall survival rate of metastatic CRC patients (late-stage (IV)) is only 4~12% [5].

Recent advances in CRC research have greatly expanded our understanding of the cellular and molecular bases of CRC carcinogenesis and metastasis and thereby considerably improved the survival of CRC patients [6–11]. Combined with high-throughput sequencing, some studies even challenge prevailing models by identifying somatic mutations and clonal dynamics in normal healthy tissues [12–16]. In addition, it was previously widely accepted that there was a sequential progression in tumors, in which primary tumor first seeds lymph node metastases, and then lymph node metastases further seed distant metastases. However, recent studies showed that subclones of lymph node and distant lesions within the same patient may have independent origins through 20–43 hypermutable polyguanine repeats of formalin-fixed and paraffin-embedded samples [7]. However, most of these genomic and transcriptomic profiling studies have characterized bulk tumor tissues or just studied single omics features. The intratumoral heterogeneities and relationships among different omics of CRC have not been systematically investigated [17–19]. For example, single-cell genome sequencing allowed us to trace the clonal lineages from primary tumor to metastasized tumor from the same patient. However, single-cell genome sequencing cannot offer us clues about the phenotypic changes within the same lineage of tumor cells during the metastatic process. In other words, even if we can get the point mutation information from single-cell genome sequencing analysis, we still do not know what are the phenotypic and transcriptomic consequences of the mutations. On the other side, single-cell RNA sequencing allows researchers to compare the gene expression differences between primary and the metastatic tumors. But these transcriptome differences may be just a reflection of the clonal extinction or expansion during the metastatic process. Approaches at single-cell resolution and multiomics levels can help us better understand metastasis process of CRC and identify potential therapeutic targets for metastatic CRC. A deeper understanding of tumor transcriptomic heterogeneities and integration of different genomic profiles, such as CNVs, somatic mutations, and gene expression, can greatly advance our understanding of the cause, classification, progression, and phenotype-genotype relationship of CRC. Furthermore, tracing the dynamic changes in the genome and transcriptome of cancer cells during CRC metastasis is of vital importance for the treatment of metastatic CRC. Thus, we performed a single-cell transcriptomic survey, whole genome sequencing (WGS) and multi-region whole exome sequencing (WES) of 12 metastatic CRC patients to investigate multiomics changes during tumorigenesis of CRC. Through single-cell RNA-seq data, we find that PPAR signaling pathway probably can serve as a potential target for CRC treatment. We recapitulated the process of tumor metastasis and changes in clonal compositions by tracing mitochondrial mutations. Furthermore, we delved into the relationships between point mutations and gene expression patterns by integrating single-cell targeted cDNA Sanger sequencing and single-cell RNA-Seq data. Our study provides valuable genomic and transcriptomic data and new insights for the molecular mechanism studies of colorectal cancer.

## Methods

### CRC specimen collection

Samples were taken from primary tumor and adjacent normal colon (at least 10 cm away from the tumor border) tissues immediately after specimen resection. For patients #3–#5 and #7–#9, multi-region sampling, including margin and center regions, was performed to investigate intratumoral heterogeneities. At the same time, preoperative imaging-suspected liver, lymph node, and omental metastases were sampled and were further confirmed by postoperative pathological examination. Each sample was cut into 3 parts, one for single-cell capture, one for bulk sequencing, and one for paraffin-embedding. In total, we sequenced samples from 12 patients. As for tumor tissues, we performed single-cell RNA-seq for 9 patients (patients #1–#9) and WES for patients #7–#9 as well as another 3 patients (patients #10–#12). One patient (patient #1) received two cycles of systemic chemotherapy before surgery, and another patient (patient #5) was classified as having a microsatellite instability-high (MSI-H) tumor caused by failure of the DNA mismatch repair (MMR) system. In total, we generated high-precision single-cell RNA-Seq profiles of 9120 individual cells from 11 adjacent nontumor colonic mucosa (N), 9 primary tumors (PTs), 12 matching lymph node metastases (LyMs), 7 liver metastases (LMs), 3 omentum metastases (OMs), and 1 liver normal tissue (LN).

### Tissue dissection and single-cell capture

Fresh samples were processed for single-cell collection within 2 h. The submucosa and muscle layers of normal colon tissues and the adipose tissue and visible blood vessels of the primary tumor (PT) and metastasis site were removed for further experimental steps under the microscope. The processed samples were washed with 1× PBS for six times until the supernatant was clear. Then, the samples were cut into small pieces with surgical scissors. Collagenase/Dispase (Roche, 10269638001) at 2 mg/mL was used to digest the washed samples into single-cell suspensions at 37 °C for 60–70 min. We then employed a 40-μm strainer (BD Falcon, 352340) to filter out cell aggregates, after which the cell suspension was centrifuged at 800*g* for 5 min. The cell pellet was subsequently washed twice with DMEM containing 10% FBS. Finally, single cells in good condition were picked and transferred to single-cell lysis solution with a mouth pipette. To make sure that each time only a single cell was picked up, first we made three 20 μl 1% BSA drops in a 6-cm dish. Then 5–10 μl cell suspension (the volume depends on cell density) was added into drop #1. Under microscopy, we could clearly identify single cells from the doublets, triplets, or cell clusters. We then picked up about 30 bright spherical single cells to drop #2 using a mouth pipette under microscopy, and this step was used to wash the picked cells. Then we further picked up single bright spherical cells from drop #2 to drop #3, which is to further wash these single cells, and after the wash steps, we got a clear drop that contains about 20 well dispersed single cells in drop #3. At this time, we picked only one single cell from drop #3 to each 0.2-ml PCR tube. Through these processes, we can effectively eliminate the potential doublets, triplets, or cell clusters.

### Patient-derived organoid culture

Surgically resected tumor tissues and normal tissues were isolated into small pieces and were then digested into single-cell suspensions by collagenase (type II and type IV; Invitrogen, 17101015 and 17104019). After digestion, cancer cells were passed through a 40-μm cell strainer (Corning, 352340). Normal mucosa was first stripped off from the muscle layer and was cut into small pieces. After at least three times of washing, the fragments were transferred into PBS containing EDTA and were shaken vigorously at 4 °C for 15 min. The crypts that were shaken off were collected by centrifuge. The suspensions were centrifuged and then resuspended in Matrigel and plated in a 24-well cell culture dish. After 20 min of solidification in a humidified incubator at 37 °C, culture medium was then added. The composition of the culture medium was as follows: Advanced DMEM/F12, 2 mM GlutaMAX, 100 U/mL penicillin/streptomycin, 0.5 μM A83-01 (R&D systems, 2939), 1× B27 (Invitrogen, 17504044), 10 nM prostaglandin E2 (R&D systems, 2296), 5 μM SB202190 (Sigma, S7076), 10 mM nicotinamide (Sigma, N0636), 500 ng /mL R-spondin (R&D systems， 4645-RS), 1 mM N-acetylcysteine (Sigma, A9165), 50 ng/mL EGF (Peprotech, AF-100-15), 10 nM gastrin I (Sigma, G9145), 100 ng/mL Wnt3A (R&D systems, 5036-WN), 100 ng/mL Noggin (Peprotech,120-10C). Notably, 10 μM Y-27632 (Selleck, S1049) was supplemented in the first week. Tumor organoid culture medium contained the above components without Wnt3A. The media were changed every 2 days. Organoids were passaged every 1–2 weeks and were maintained for long-term culture.

### Single-cell RNA-seq library construction

The high-precision single-cell RNA sequencing protocol we used was a modified STRT-Seq protocol. In the reverse transcription (RT) step, we used a predesigned RT primer (5′-TCAGACGTGTGCTCTTCCGATCTXXXXXXXXNNNNNNNNT25-3′) to tag the cDNAs of each single cell with an 8-bp barcode sequence (X8) [20]. The 8-bp random unique molecular identifier (UMI) (N8) in the RT primer was used to prevent PCR bias. The Template Switching Oligonucleotide (TSO) primer used in SMART-seq2 was used in our protocol [21]. After RT, the cDNA was pre-amplified with the IS primer (10 μM, 5′-AAGCAGTGGTATCAACGCAGAGT-3′), which paired with the TSO primer and the P2 primer (10 μM, 5′-GTGACTGGAGTTCAGACGTGTGCTCTTCCGATC-3′), which paired with the barcode primer. Then the pre-amplified cDNAs with different barcodes were pooled together for the following steps. After purification with AMPure XP beads (Beckman, A63882), we further amplified the pooled cDNAs by using the IS primer (5′-AAGCAGTGGTATCAACGCAGAGT-3′) and biotin primer (5′-/Biotin/CAAGCAGAAGACGGCATACGAGAT/Index/GTGACTGGAGTTCAGACGTGTGCTCTTCCGATC-3′), after which the biotin-tagged cDNAs were fragmented into 300-bp fragments. Then, we used Dynabeads® MyOne™ Streptavidin C1 (Invitrogen, 65001) to enrich the biotin-tagged ends. KAPA Hyper Prep Kits with the PCR Library Amplification/Illumina series (KAPA, KK8054) were used to construct the library. The library was sequenced with an Illumina HiSeq 4000 platform for 150-bp paired end reads. With the modified STRT-seq, we only obtained sequence information from the 3′ ends of the mRNAs.

### Sanger sequencing with the single-cell cDNA product

The mutation sites were detected by Sanger sequencing. The cDNAs used for mutation detection were the remaining product from single-cell RNA sequencing. While conducting the pooling process of single-cell RNA sequencing, half of the cDNAs were pooled together for further library construction, the remaining half of the cDNAs were preserved for Sanger sequencing. To verify the accuracy of mutation sites detected by Sanger sequencing and eliminate the possibility of contaminations, 48 cells (KRAS P9_LM1_Batch3) from one patient were sequenced twice (Additional file 1: Table S6).

The sequences of primers used can be found in Additional file 1: Table S10.

### Laser capture microdissection

Considering the low purity of tumor tissue in lymph node metastases (LyMs), tumor areas were extracted from tissue sections by laser capture microdissection (LCM). Paraffin-embedded LyMs were sectioned into 5–10 consecutive 8-μm-thick slides which were then attached onto PEN membrane slides (Leica,1150515). After H&E staining, tumor areas were confirmed by two independent pathologists. Then tumor areas were obtained by LCM (Leica LMD7000 Microsystem). Tumor patches were pooled together for genomic DNA extraction using the GeneRead DNA FFPE Kit (Qiagen, 180134) and then the extracted DNA was used for WGS.

### Whole genome sequencing

The extracted genomic DNA was fragmented into 300-bp fragments via sonication. Libraries were then constructed according to the instructions of the manufacturer of the KAPA Hyper Prep Kit (KAPA, KK8054).

### Whole exome sequencing

The extracted genomic DNA (approximately 200 ng) was fragmented into 150–200 bp fragments through sonication, followed by end-repairing. Fragmented DNA was then ligated with a predesigned barcoded adaptor in which 3 bp barcode sequence was added to the NEB adaptor sequence. Therefore, the DNA ligated with different barcoded adaptors could be pooled together for further steps. Barcoded adaptors ligated DNA was further amplified with the NEB index primer, universal primer, and 2× KAPA HiFi HotStart ReadyMix (KAPA, KK8054) for 4 cycles. SureSelectXT Human All Exon v6 was used to capture the exome regions of different barcode pooled libraries. The library construction and high-throughput sequencing of these captured exome sequences were the same as WGS.

### Bulk RNA sequencing

RNA was first extracted using the RNeasy Mini Kit (Qiagen, 74104), and then mRNA was reversed transcribed and amplified. Approximately 50 ng of amplified cDNAs were used to perform library construction following the instructions of the TruePrepTM DNA library Prep Kit V2 (Vazyme Biotech, TruePrepTM DNA library Prep Kit V2).

### Hematoxylin-eosin (H&E), immunohistochemical (IHC) staining, and immunofluorescence staining

Fresh tissues were fixed with 10% neutral buffered formalin and then embedded in paraffin. Next, the paraffin-embedded tissue blocks were sectioned into 5-μm-thick slices. H&E staining was performed on these 5-μm sections. For IHC staining, the sections were boiled in 0.01 M citrate buffer (pH 6.0) for 30 min for antigen retrieval, treated with 3% hydrogen peroxide solution to block endogenous peroxidase activity, and then blocked with 10% BSA for 1 h at room temperature. After incubation with primary antibody and appropriately diluted secondary antibody, the slices were visualized using DAB substrate liquid and photographed with NanoZoomer SQ.

For immunofluorescence staining, slices were stained with primary antibodies at 4 °C overnight and then incubated with the fluorochrome-conjugated secondary antibody for 1–2 h at room temperature in the dark. After rinsing for three times with PBS, the slices were counterstained with DAPI. Images of all tissues were visualized via confocal microscopy. Positive cell ratios were calculated with a PerkinElmer Launches Mantra™ Quantitative Pathology Imaging System. All images were examined and analyzed by two independent pathologists.

### Cell apoptosis assay and cell cycle analysis

For apoptosis assay, organoids were first digested into single-cell suspensions and then centrifuged at 800 g for 5 min. Then cells were suspended with 400 μL binding buffer containing 5 μL of Annexin V-FITC and 10 μL of PI staining solution (BestBio, BB-4101). After staining for 5 min, flow cytometry was used to distinguish dead and apoptotic cell populations. As for the cell cycle analysis, approximately 400,000 cells per well were first implanted into 6-well plates for 12 h and then drugs were added. Next, FxCycle™ PI/RNase Staining Solution (Life Technologies, F10797) was used to perform cell cycle analysis. At least three replications were performed for each experiment.

### Organoid drug response assay

Through performing GO enrichment analysis on DEGs between normal epithelial cells and tumor cells, we showed that tumor-specifically overexpressed genes were involved in the PPAR signaling pathway. In addition, research about the role of PPAR signaling pathway in CRC tumorigenesis remains controversial. Therefore, we focus on the PPAR signaling pathway for further analysis. FH535 (Selleck, S7484), XAV939 (Selleck, S1180), and GW9662 (Selleck, S2915) were purchased from Selleck and were dissolved in DMSO in aliquots of 100, 20, and 100 mM respectively. The tumor organoids were gently digested and then planted into a 96-well cell culture plate by adding 10 μL of Matrigel droplets containing about 3000 cells to each well. Then 3 days later, inhibitors were added to the culture medium of organoids and for each drug treatment, three replicate wells were set. After 5 days of inhibitor treatment, the organoid cell viability under different treatment conditions was measured by the CellTiter-Glo 3D reagent (Promega, G9683) and luminescence was measured with GM2000 GloMax® Navigator (Promega, GM2000). For each inhibitor, the results were normalized by dividing the cell viability of the negative control (0.1% DMSO). Then, the tumor inhibition curve of a certain inhibitor was fitted with Prism 7 software. As for the drug combination experiment, organoids were resuspended with 30 μl Matrigel and plated on a 24-well culture plate. After 2 days culture, different concentrations of XAV939 and GW9662 were used to treat tumor organoids simultaneously. Cells were collected after 5days of treatment to measure the cell viability using CellTiter-Glo 3D reagent (Promega). All experiments were processed with at least three technical replicates. FH535 is an inhibitor of Wnt/β-catenin signaling and dual antagonist of PPARγ/δ activity. It inhibits β-catenin and GRIP1 recruitment to PPARγ and δ. Another Wnt signaling pathway specific inhibitor, XAV939, selectively inhibits Wnt/β-catenin through tankyrase 1/2 inhibition. So both of these two inhibitors inhibit Wnt signaling through Wnt/β-catenin, but FH535 directly inhibits Wnt/β-catenin while XAV9393 indirectly inhibit it. For each concentration, three technical replicates (at the same time) were set. We repeated the whole experiment twice (two batches at different time) and both got similar results.

### Whole-mount immunofluorescence

The organoids were first washed twice with PBS and fixed in 4% paraformaldehyde for 1 h. Then the following steps were performed: (1) permeabilizing the organoids at room temperature for 30 min using 0.5% Triton X-100, (2) transferred the organoids into blocking buffer and incubated overnight at 4 °C, (3) incubating the organoids with primary antibodies which were diluted with blocking buffer at 4 °C overnight, (4) incubating the organoids with secondary antibodies for 2 h at room temperature, and (5) staining the organoids with DAPI (1:500 diluted) for 10 min. The immunofluorescence was visualized using a Nikon A1RSi+ confocal microscope. Antibodies used for immunofluorescence are shown in Additional file 2: Table S10.

### Self-renewal and differentiation potential of SOX9/MKI67-positive cells

In total, three organoid cell lines (patient #1, O#H and O#S) were derived to verify the self-renewal and differentiation potential of *SOX9/MKI67*-positive cells. These organoid cell lines were established as the steps mentioned above (patient-derived organoid culture section). As for Organoids O#H, we first performed whole-mount immunofluorescence of SOX9 and MKI67 on short-term (~1 week) and long-term (~2 months) cultured organoids to explore whether *SOX9/MKI67*-positive tumor cells have the self-renewal potential*.* To further explore the differentiation potential of *SOX9/MKI67*-positive cells, we thawed passage-11 O#H organoid and further cultured it for another 1 month. Then cells were digested into single cells for single-cell RNA sequencing. In addition, we also performed whole-mount immunofluorescence of CA2 on 1-month cultured O#H organoids. In order to rule out the possibility that the increase ratio of *SOX9/MKI67*-positive cells in vitro is due to the death of differentiated cells in vitro, we further performed single-cell RNA-seq on five single organoid spheres of O#S. Specifically, five single tumor organoid spheres of O#S were picked into drops of TrypLE (Invitrogen, 12605028) and digested into single-cell suspensions at 37 °C respectively.

### Whole exome sequencing data analysis

Duplicate marked mapping reads were attained for WGS analysis, and then GATK (Genome Analysis Toolkit, Version 3.8) and Mutect2 were used to call the somatic mutations [22, 23]. The whole pipeline could be separated into four steps: (1) local realignment around indels by RealignerTargetCreator and IndelRealigner, (2) base quality score recalibration with BaseRecalibrator and PrintReads, (3) somatic variant calling by Mutect2 with blood as a control, and (4) variant annotation with SnpEFF [24]. Codes can be found at the GitHub (https://github.com/WRui/Metastatic-Colorectal-Cancer) [25].

### Single-cell RNA-Seq data pre-processing and read mapping

Paired end sequencing reads were split according to the cell-specific barcodes in reads 2, and the UMI sequences in reads 2 were attached to reads 1. Then, quality control pipelines were applied to remove low-quality and adapter-contaminated reads. The parameters and corresponding codes can be found at the GitHub website (https://github.com/WRui/Post_Implantation/tree/master/scRNA_UMI) [26]. Next, TopHat (version 2.0.14) was used to map the cleaned reads to the human genome (hg19), and only uniquely mapped reads were retained [27]. HTSeq was employed to estimate the abundance of the transcripts by counting the uniquely mapped reads for each gene, and reads with duplicated UMIs for each gene were excluded [28]. Finally, the abundance of a gene was normalized to TPM.

### Removal of low-quality cells and low-abundance genes

**Fig. 1:**
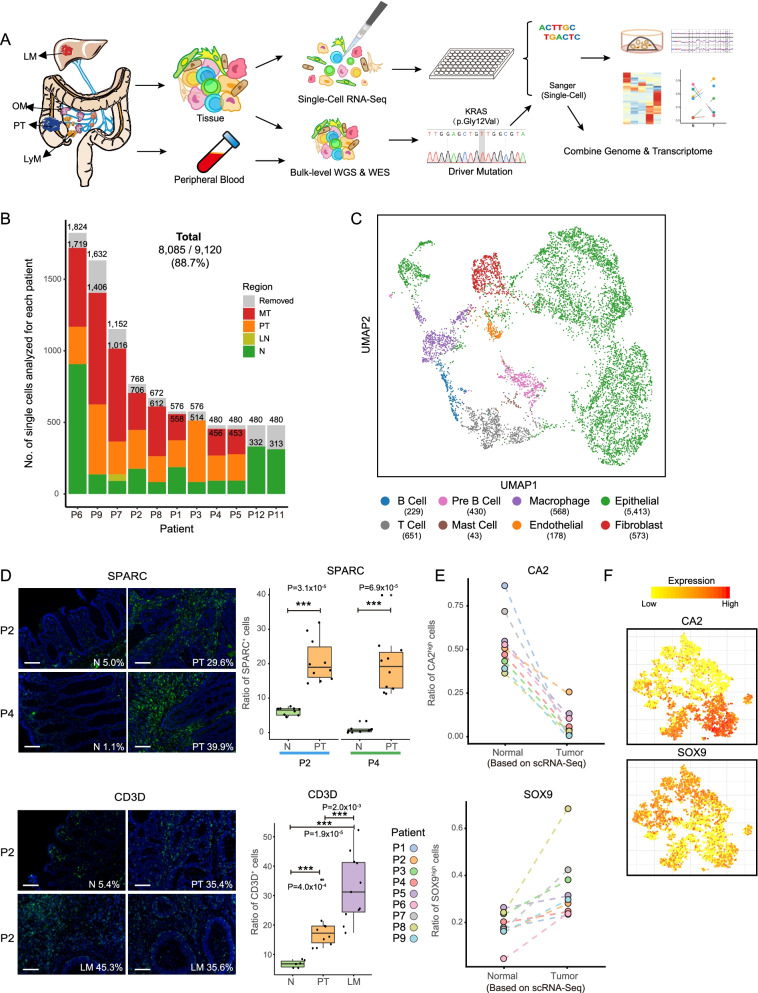
Single-cell transcriptome analysis of colorectal cancer. **A** The workflow illustrates the strategy for cell collection from matching adjacent normal tissues and primary and metastatic colorectal tumors for single-cell RNA-Seq, single-cell cDNA Sanger sequencing, and bulk level whole genome sequencing and whole exome sequencing. **B** Bar plot showing the number of cells collected from each patient. Patients are ordered according to the total number of cells. Color represents cell origin. Low-quality cells are removed with strict criteria. Only cells that expressed at least 1000 genes and showed second maximum pairwise Pearson correlations greater than 0.6 were retained for further analysis. Finally, 8085 cells (88.7%) were retained for subsequent analyses. The details can be found in the “Methods” section. PT: primary tumors. LN: adjacent normal tissue collected from liver. N: adjacent normal tissues. MT: metastatic tumors, including lymph node metastasis, liver metastasis, and omentum metastasis. Removed: cells that have not passed the quality control and not used for subsequent analysis. **C** UMAP plot of cell clusters. Cell types were identified base on the regulon activity matrix and then visualized by UMAP. Cells were colored according to annotated cell types. According to their expression of known marker genes shown in Supplementary Figure 1B, we annotated these clusters as epithelial cells, endothelial cells, fibroblasts, T-cells, B-cells, pre-B-cells, macrophages, and mast cells. Associated with Supplementary Figure 1A and Supplementary Figure 1C-D. Most of the immune cells, fibroblast, and endothelial cells come from the tumor area. **D** Immunofluorescence staining of the shared endothelial and fibroblast marker SPARC and T-cell marker CD3D in adjacent normal tissues, primary tumor, and liver metastasis. The boxplot shows the SPARC- or CD3D-positive cell ratio in different regions (N: adjacent normal tissue; PT: primary tumor; LM: liver metastasis). Scale bar, 100μm. **E** The dot plot shows the ratio of cells that highly expressed enterocyte marker (*CA2*) and intestinal stem cell marker (*SOX9*) in normal and tumor regions for each patient. **F** The expression levels of enterocyte marker (*CA2*) and intestinal stem cell marker (*SOX9*) were projected on epithelial cells tSNE maps. Colors from yellow to red represent expression levels from low to high

Stringent quality filter criteria were used to filter out low-quality cells. Only cells that expressed at least 1000 genes and showed second maximum pairwise Pearson correlations greater than 0.6 were retained for further analysis. Only genes that showed a log_2_(TPM+1) expression value over 1 in four cells were retained for subsequent analysis. After the application of an automated quality control pipeline, 88.7% of the cells (8085 cells) were retained for subsequent analyses with an average of 3685 genes being detected in each individual cell (Fig. 1B and Additional file 1: Table S1). Since we traced the tumor clonal structure based on the mitochondrial mutations, we did not filter cells based on the ratio of reads mapped to mitochondria. The median ratio of reads that mapped to mitochondria is just 12.5% which reflected the reasonable quality of our single-cell RNA-seq dataset.

### Nonlinear dimensional reduction on the transcriptome expression matrix

Nonlinear dimensional reduction (tSNE) was analyzed on our filtered expression data using the scater package by executing the “plotTSNE” function [29].

### Verification of DEGs of BRAF^V600E^ and BRAF^600WT^ in TCGA-COAD dataset

The somatic mutation data of TCGA-COAD cohort were firstly downloaded from xena website. Then we grouped samples according BRAF 600 AA and separated into two groups: BRAF^V600E^ and BRAF^600WT^. According to single-cell RNA-seq result, we found that *L3MBTL2* is highly expressed in BRAF^V600E^ cells, regardless of whether the cells were from primary or metastatic tumors. In order to verify that the phenomenon of high expression of *L3MBTL2* in BRAF^V600E^ cells is widespread, we analyzed the TCGA-COAD bulk RNA-seq data and found that the expression of *L3MBTL2* indeed increased significantly in BRAF^V600E^ samples.

### Identification of DEGs and biomarker clustering

The “FindAllMarkers” function with the parameter "thresh.test=1.5" in the Seurat R package (v3.0.2) was used to identify DEGs associated with specific features (more than three, features here represent different cell clusters) [30]. As two features, the “FindMarkers” function was employed to obtain the DEGs.

### Single-cell transcription factor regulatory network construction and clustering

The SCENIC package was employed to establish a gene regulatory network and to simultaneously group our single cells using the inferred networks [31]. Following the SCENIC manual, we obtained a filtered version of a binary activity matrix of regulons. Then, unsupervised hierarchical clustering was employed to cluster the cells based on the binary activity matrix. Cells were grouped into eight main clusters and DEGs were identified for each cell cluster. According to DEG lists and well-known cell-type marker genes, these 8 clusters were annotated as epithelial cells (*EPCAM*, *CDH1*, *CDX2* and *MUC2*), fibroblasts (*VIM*, *THY1*, *COL1A1*, and *COL1A2*), endothelial cells (*VIM*, *CDH5*, and *PECAM1*), pre-B (*PTPRC*, *CD79A*, and *CD79B*) cells, B-cells (*PTPRC*, *CD79A*, *CD79B*, and *MS4A1*), T-cells (*PTPRC*, *CD3D*, *CD3E*, *CD3G*, and *CD8A*), mast cells (*PTPRC* and *KIT*), and macrophage cells (*PTPRC*, *CD163*, *CD68*, and *FCG2R*). Due to the limitation of the figure numbers, we only showed expression pattern of one cell-type-specific gene for each cell type in Additional file 2: Fig. S1D. After identifying cell clusters, cells were visualized with UMAP in the Seurat package (v3.0.2) by running RunUMAP function with setting dims=1:10.

### CNVs inferred based on single-cell RNA-Seq data

Single-cell RNA-Seq data were used to infer CNVs according to a previously published method [32]. We used the published software inferCNV to infer CNVs (https://github.com/broadinstitute/infercnv) [32]. It showed that except patient #5 that is classified as MSI-high tumor, almost all epithelial cells collected from tumor tissues contained CNVs and epithelial cells collected from adjacent normal tissues exhibited normal diploid state. In addition, according to the clustering results shown with UMAP, epithelial cells collected from adjacent normal tissues and tumor tissues clustered separately. Therefore, in our study, we defined epithelial cells collected from tumor tissues as tumor cells, while epithelial cells collected from normal tissues as normal epithelial cells.

### CNVs confirmed via bulk whole genome sequencing

To confirm the CNV results at the DNA level, we sequenced the whole genome of bulk samples from the corresponding single-cell RNA-Seq dataset. First, low-quality reads and adapter-contaminated reads were removed. Then, BWA was employed to map the cleaned reads to the human genome (hg19). The whole genome was divided into 10-M windows, and the total reads located in each window were calculated and normalized by the total reads of each sample. Next, for each window, the reads were scaled by the average read depth of all samples. Finally, dot plots were used to visualize the CNV patterns.

### Mitochondrial mutation calling with single-cell RNA-Seq data

The output bam files of TopHat were used for subsequent mitochondrial SNP calling by GATK according to online suggestions for RNA-Seq data [23]. The SNP calling pipeline can be found at GitHub (https://github.com/WRui/Metastatic-Colorectal-Cancer) [25].

### Tumor phylogenetic reconstruction

To explore the relationships between different tumor sites, only mutations that shared by at least two samples were used for phylogenetic tree construction. The R package “ape” was used to construct the phylogenetic tree [33]. The distances between different tumor regions were first calculated with the “dist.gene” function and then the phylogenetic tree was constructed with function the “nj”. Finally, the unrooted trees were displayed by performing the “plot.phylo” function.

## Results

### Landscape of transcriptomic heterogeneities in CRC

To investigate the transcriptomic heterogeneities and cellular diversities of CRC, we generated high-precision RNA-Seq profiles of 9120 single cells from 11 adjacent nontumor colonic mucosa (N), 9 primary tumors (PTs), 12 matching lymph node metastases (LyMs), 7 liver metastases (LMs), 3 omentum metastases (OMs), and 1 liver normal tissue (LN) (Fig. 1A, B, Additional file 1: Table S1). After stringent filtration, 8085 cells (88.7%) were retained for further analyses (Fig. 1B, Additional file 2: Fig. S1A-B).

To accurately explore the diversity of cell types in CRC, we clustered these single cells based on their transcription factor regulatory networks and eight main cell clusters were identified (Fig. 1C and Additional file 2: Fig. S1C-D). Furthermore, we found that compared with other cell types, tumor epithelial cells showed highest differences with normal epithelial cells from matched adjacent normal tissues as expected. Normal epithelial cells from different patients were similar to each other, while tumor epithelial cells from different patients were separated from each other on UMAP (Additional file 2: Fig. S1E-F).

Next, we further explored the cell-type composition changes between different sites (Fig. 1D and Additional file 2: Fig. S1G-H). A diverse and complex microenvironment was revealed in PTs as well as LyMs, LMs, and OMs, with higher proportions of T-cells, B-cells, and macrophages and decreased proportions of epithelial cells in tumors than in adjacent normal tissues. In addition to the number of immune cells, the number of fibroblasts also increased in tumor tissues (Additional file 2: Fig. S1H). The enrichment of T-cells and fibroblasts/endothelial cells in tumor tissues was verified through immunofluorescent staining of CD3D and SPARC, respectively (Fig. 1D and Additional file 1: Table S10).

### Enrichment of intestinal progenitor cell and Paneth cell markers in tumor epithelial cells

As shown in Fig. 1C and Additional file 2: Fig. S1C, we found that there were dramatic transcriptome-level differences between tumor and normal epithelial cells. We further explored these differences by comparing their epithelial cell-type composition and transcriptomic features.

Globally, we found that normal epithelial cells consisted of mainly enterocytes and goblet cells (Fig. 1E, F and Additional file 2: Fig. S2A-C). In contrast, tumor cells were more likely to express Paneth cell markers and stem/progenitor cell markers and exhibited more mesenchymal-like features (Fig. 1F and Additional file 2: Fig. S2D-E). Few cancer cells expressed marker genes of differentiated cell types such as *CA2* (enterocyte marker) and *MUC2* (goblet marker) (Fig. 1E, F and Additional file 2: Fig. S2B-C).

**Fig. 2:**
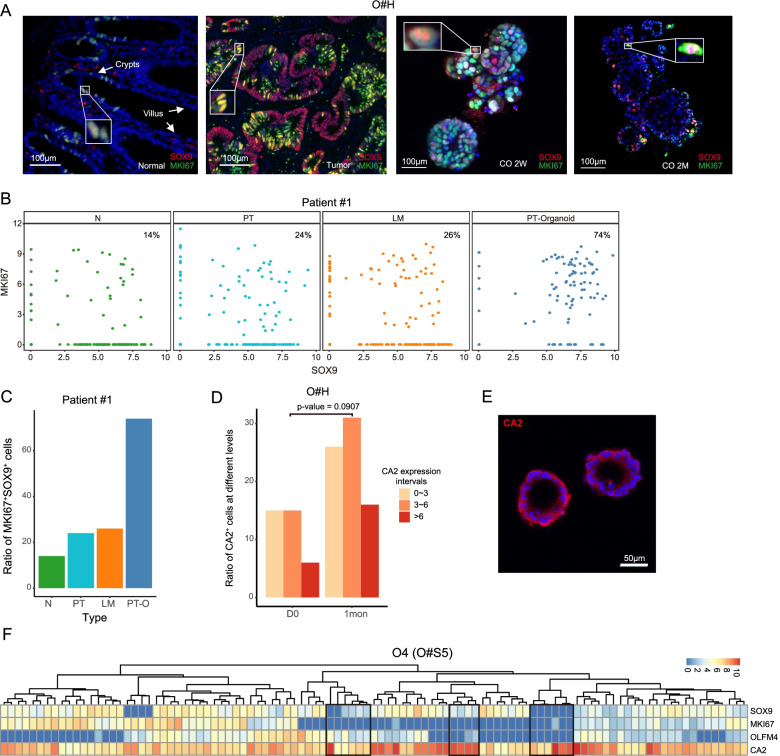
*SOX9/MKI67*-positive cells may have potential of self-renewal and differentiation. **A** Immunofluorescence staining of cell proliferation marker (MKI67) and intestinal stem/progenitor cell marker (SOX9) for adjacent normal tissue and tumor tissue in vivo as well as patient-derived cancer organoids that were cultured for 2 weeks and 2 months. CO: cancer organoid. 2W: 2 weeks. 2M: 2 months. Scale bar, 100 μm. **B** Dot plot showing the expression level of MKI67 and SOX9 of patient #1. Colors represent cells that collected from different regions. N, normal region. PT, primary tumor. LM, liver metastasis. PT organoid, in vitro cultured organoid that are derived from the primary tumor. The proportion of double positive cells is written in the upper-right corner of each dot plot. **C** Bar plot shows the ratio of SOX9/MKI67 double positive cells of patient #1 from different regions. N, normal tissue; PT, primary tumor; LM, liver metastasis; PT organoid, primary tumor-derived organoid. **D** The bar plot shows the ratio of cells that expressed different level of enterocyte marker *CA2*. D0 represents thawed tumor organoid, and 1mon represents tumor organoid that is cultured for 1 month. *P*-value was calculated through *t*-test. The color of the bar represents the expression levels (log_2_(TPM+1)) of CA2. **E** Immunofluorescence staining of enterocyte marker CA2 on long-term cultured tumor organoid (O#H). Scale bar, 50μm. **F** Heatmap shows cell-type specific genes expression in one of five tumor organoids of one patient (O#S5). Colors from blue to red represents expression level (log_2_(TPM+1)) from low to high. The black boxes highlight cells that do not express or low express stem/pluripotency markers (*SOX9*, *OLFM4*, *LGR5*, *ALCAM*, *LRIG1*, and* MKI67*) but highly express differentiated marker *CA2*

Hence, we further explored the cell-type compositions for each patient by calculating the ratio of cells expressing typical intestinal cell markers. We found that all patients showed a decreased proportion of *CA2*-positive (enterocyte marker) cells in tumor tissues, which was verified by immunohistochemical (IHC) staining (Additional file 2: Fig. S2B). In addition, the *MUC2*-positive (goblet marker) cells were also depleted in tumor tissues compared to adjacent normal tissue, which was also verified by IHC staining (Additional file 2: Fig. S2C). On the other hand, the expression of marker genes for other cell types, such as *LYZ* (Paneth cell marker) and *SOX9* (intestine progenitor cells), were enriched in tumor epithelial cells and *LYZ* expression was further verified by IHC staining (Additional file 2: Fig. S2D). These results revealed that intestinal stem cell-like cells were enriched in tumor epithelial cells. Through immunofluorescent staining of SOX9 (intestinal stem/pluripotency marker) and MKI67 (marker of proliferation), we found that stem cell-like and actively dividing cells were located only in crypt regions of adjacent normal tissue whereas nearly all tumor epithelial cells expressed SOX9 or MKI67 in vivo (Fig. 2A).

We further established a patient-derived organoid culture system to explore whether these stem cell-like cells had the potential for self-renewal and differentiation into more mature cell types. We first compared the ratio of *SOX9*/*MKI67*-positive cells in normal and tumor tissues in vivo and primary tumor-derived organoid in vitro. The ratio of *SOX9/MKI67*-positive cells in organoids were much higher than their in vivo counterparts (Fig. 2B,C). It indicated that *SOX9/MKI67*-positive cells may have growth advantage in vitro and have the potential of self-renewal. Next, we performed immunostaining on short- and long-term cultured tumor organoids (O#H) (Fig. 2A). For tumor-derived organoids cultured for 2 weeks, majority of the cells expressed SOX9/MKI67 (SOX9^+^ 41.6%, MKI67^+^ 77.5%, SOX9^+^MKI67^+^ 37.7%), but after long-term culture (2 months), less cells expressed SOX9 or MKI67. Next, we performed single-cell RNA-seq on these organoid cells and the organoid cells being cultured for another 1 month from the same patient (O#H) (Fig. 2D,E). It showed that after long-term culture, the proportion of cells highly expressing the differentiation marker gene *CA2* increased significantly and immunofluorescence staining also confirmed that majority of the organoid cells expressed CA2 after long-term culture (Fig. 2D). To further verify the differentiation potential of *SOX9/MKI67*-positive cells, we performed single-cell RNA-seq on five individual spheres of organoids from one patient (O#S5) (Fig. 2F and Additional file 2: Fig. S3). Generally, the cells in an individual organoid sphere were derived from a few original cells, or even a single cell (Additional file 2: Fig. S3A). Therefore, by analyzing mitochondrial mutations, we can trace the lineage of cells in an individual organoid (Fig. 2F and Additional file 2: Fig. S3B-C). We found that within the same organoid sphere, there were cells of different cell states with the same mitochondrial mutations, both stem cell-like cells and differentiated cells, which indicates that *SOX9/MKI67*-positive cells have the potential to differentiate into other mature cell types during culture process in vitro. Taken together, these results indicated that *SOX9/MKI67*-double positive cells had the potential for self-renewal and could differentiate into multiple more mature cell types in culture, which are characteristics of stem cell-like cells.

### Transcriptomic differences between tumor and adjacent normal epithelial cells

**Fig. 3:**
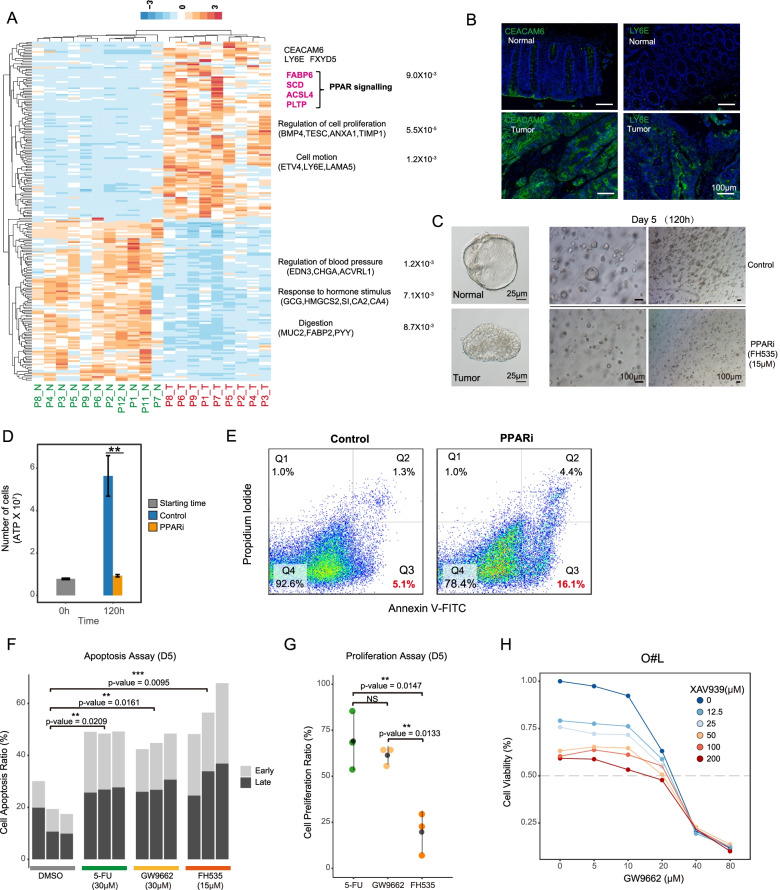
Transcriptomic differences between adjacent normal tissues and tumor tissues. **A** Heatmap showing the differentially expressed genes (DEGs) between adjacent normal and tumor tissues in each patient. Colors from blue to orange represent the expression level from low to high. The enriched gene ontology (GO) terms and *p*-value were showed in the right side of the heatmap. The full list of DEGs is summarized in supplementary table 2. **B** Immunofluorescence staining of CEACAM6 and LY6E in adjacent normal tissue and primary tumor tissue for another two patients. Both CEACAM6 and LY6E are highly expressed by tumor epithelial cells. Scale bar, 100 μm. **C** Bright-field images of patient-derived normal and tumor organoids (left panel). Representative bright-field images of PPAR inhibitor (FH535)-treated and untreated organoids (right panel). **D** Cell viability assay of PPAR signaling pathway inhibitor (FH535)-treated and untreated organoids. ** represents *p*-value < 0.05. **E** Cell apoptosis assay of PPAR inhibitor (FH535)-treated and control organoids. The results of fluorescence-activated cell sorting (FACS) are shown. Q2 represents late apoptotic cells, Q3 represents early apoptotic cells, and Q4 represents living cells. **F** Bar plot shows the ratio of apoptotic cells, light gray represents early apoptotic cells, and dark gray represents late apoptotic cells. *P*-values are calculated by *t*-test. ** represents *p*-value < 0.01. **G** Dot plot showing the proliferation ratio of cells. Different colors represent different drug treatment. 5-FU: 30 μM; GW9662: 30 μM; FH535: 15 μM. *P*-values are calculated by *t*-test. ** represents *p*-value < 0.01. NS represents not significant. There are 3 replications for each treatment. **H** The line plot showing the cell viability of tumor organoid (O#L) under different concentrations of drugs. The *x*-axis represents the concentration of PPAR inhibitor GW9662, and the *y*-axis represents the cell viability of tumor organoid with 5 days drug treatment. Colors from blue to red represent the concentration of WNT inhibitor XAV939 from low to high

To eliminate the interference of nonepithelial cells, differentially expressed genes (DEGs) were identified between the normal and tumor epithelial cells (Fig. 3A). As expected, the tumor cells tended to show lower expression of differentiation genes such as enterocyte markers (*CA1*, *CA2*, and *CLCA1*) and endocrine cell markers (*PYY* and *GCG*) (Fig. 3A and Additional file 1: Table S2). Moreover, tumor cells showed lower expression of the metallothionein family genes, including *MT1H* and *MT1G* (Fig. 3A and Additional file 1: Table S2). On the other hand, tumor cells tended to show higher expression of *LY6E*, *FXYD5*, and *TGFBI* (Fig. 3A). Furthermore, higher expression levels of the tumor marker CEACAM6 were verified via IHC staining in five patients in this cohort and three additional patients (Fig. 3B and Additional file 2: Fig. S4A-B). CEACAM6 also showed higher expression levels in other CRC patients reported on the Human Protein Atlas website (https://www.proteinatlas.org/ENSG00000086548-CEACAM6). The expression pattern of LY6E was also verified in additional patients (Fig. 3B).

### PPAR inhibitor can inhibit the growth of CRC organoids

Of the DEGs between normal and cancer tissues, we found that PPAR signaling pathway-associated genes were strongly upregulated in tumor epithelial cells compared to normal epithelial cells (Fig. 3A and Additional file 1: Table S3). However, we found that the PPAR signaling pathway-related genes that were highly expressed in tumor epithelial cells in single-cell data were not all highly expressed in tumor tissues in TCGA-COAD data (Additional file 2: Fig. S4C). Some genes even showed the opposite trend and were highly expressed in normal tissues, such as *SCD* and *ACSL4*. PPARs are metabolic regulators that participate in the regulation of glucose and lipid homeostasis and there are three subtypes of PPARs that encoded by distinct genes [34, 35]. Although extensive studies have explored the role of PPAR in colorectal cancer through PPAR agonists or gene knockdown experiments, there are still many controversies about them. In addition, these studies were mainly performed in cancer cell lines that cultured in a 2D environment, which usually have been cultured in vitro for very long time, thus may not fully reflect the original heterogeneities of tumors in vivo [36]. To further explore the function of PPAR signaling pathway in tumorigenesis, we treated tumor-derived organoids with the drug FH535, which is a Wnt/β-catenin signaling pathway inhibitor and a dual PPARγ and PPARδ antagonist (Fig. 3C–E and Additional file 2: Fig. S5A-C). Compared with those of the untreated organoids, both the size and proliferation rate of FH535-treated organoids drastically decreased (Fig. 3C, D and Additional file 2: Fig.S5A-C). We also explored the cell apoptosis by double staining with PI and Annexin V and found that FH535-treated organoids had a drastically increased proportion of apoptotic cells (20.5% compared with 6.4%) (Fig. 3E). Since FH535 inhibits the recruitment of the coactivators GRIP1 and β-catenin to PPARγ and PPARδ, to exclude the effect of the WNT signaling pathway, we also treated organoids with the WNT signaling inhibitor XAV939 and found that neither the proliferation rate nor the morphology of the organoids showed significant changes (Additional file 2: Fig. S5A-B). Then, we treated three tumor organoid cell lines with six different concentrations of WNT inhibitor XAV939 and dual-inhibitor FH535 respectively. It showed that WNT inhibitor had quite mild anti-tumor effect, and even at high concentration (200μM), its suppressive effect is less than 50% (Additional file 2: Fig. S5D and Fig. 3H). To further verify the function of PPAR signaling pathway, we treated tumor-derived organoids with GW9662, an inhibitor specifically inhibits PPAR signaling pathway and compared its tumor inhibition effect with the clinically widely used drug 5-FU (Fig. 3F,G and Additional file 2: Fig. S5E). Inhibiting PPAR signaling pathway alone can effectively promote tumor cell apoptosis and inhibit tumor cell growth, and its effect was equivalent to 5-FU when the drug concentration is about 30 μM (Additional file 1: Fig. S5E). Notably, when PPAR and WNT signaling pathway were simultaneously inhibited by dual pathway inhibitor FH535, tumor proliferation was further reduced compared with organoids in which that only PPAR signaling pathway was inhibited (Fig. 3F,G and Additional file 2: Fig. S5D). Thus, we further explored the combination therapy effect of PPAR and WNT inhibitors at different concentrations (Fig. 3H). The result showed that when inhibiting PPAR signaling pathway with low concentration of GW9662, additional WNT inhibitors can slightly increase the tumor killing ability further. But additional WNT inhibitor has no effect when organoids were treated with high concentration of PPAR inhibitor (Fig. 3H). Together, these results indicate that PPAR signaling pathway promotes proliferation and inhibits apoptosis of colon cancer cells.

For further analysis of the downstream molecular mechanisms, we also performed RNA-Seq in both FH535-treated organoids and control organoids. We found that 299 genes were upregulated and 516 genes were downregulated after FH535-treatment (Fig. S5E and Additional file 1: Table S3). Downregulated genes were enriched for GO terms related to the cell cycle, p53 and PPAR signaling pathways, and this sequencing result is consistent with our phenotypic observations (Fig. 3D,E and Additional file 2: Fig. S5F). GW9662 is a selective PPAR antagonist for PPARγ, while FH535 is a PPARγ and PPARδ antagonist. According to RNA-seq data, PPARγ downstream genes were clearly downregulated, indicating that the tumor-suppressing effect of FH535 is mainly mediated by PPARγ [37]. These data suggest that PPAR inhibitors may have potential therapeutic values in the treatment of CRC.

### Lineage tracing by mitochondrial mutations and CNVs in individual cells

**Fig. 4:**
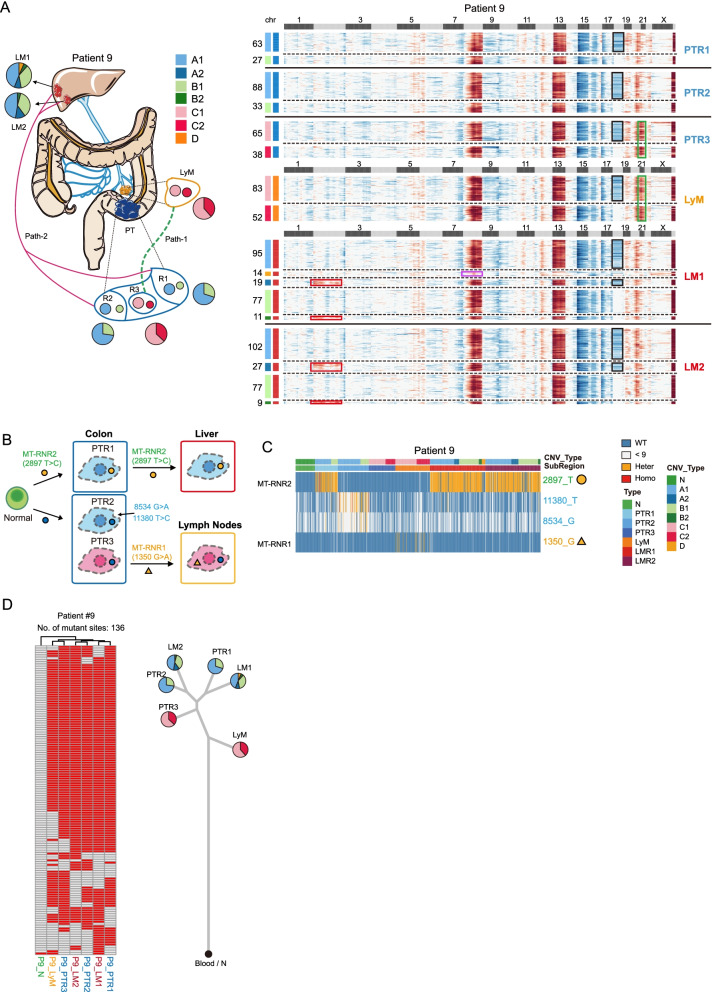
Tumor clonal architectures and tumor lineage inferences. **A** CNV pattern and sampling strategy of patient #9. Different subclones with distinct CNV patterns are found in lymph node and liver metastases. N: adjacent normal tissues. PT: primary tumors and R1-R3 represent different regions of primary tumor. LyM: lymph node metastasis. LM: liver metastasis and LM1 and LM2 represent two separated liver metastatic tumors. The pie charts reflect the proportion of different subclones in each region. Copy number gain and copy number loss are indicated with red and blue respectively. Different tissues are divided by bold black solid lines, and different clones within the same tissue block are divided by black dashed lines. The squares above the heatmap represent different chromosomes, black squares represent odd-numbered chromosomes and chromosome X, and light gray squares represent even-numbered chromosomes and chromosome Y. The squares on the left of the heatmap represent different subclones, and the same color represents the same clone. The most obvious copy number differences between different clones are highlighted by different colored boxes. Red squares in chromosome 2 show the additional CNVs of metastatic tumor (A2 and B2 subclones) compared to the primary tumor (A1 and B1 subclones). Pink square in chromosome 8 shows D subclone-specific CNV. Black squares in chromosome 18 show the A (A1 and A2) and C (C1 and C2) clones specific CNVs. Green squares in chromosomes 20 and 21 show C (C1 and C2) clone-specific CNVs. The numbers next to the heatmap show the number of cells of each subclone in different tissue blocks. **B** The diagram showing the tumor metastasis path of patient #9. The color of box represents different areas; blue, orange, and red boxes represent the primary tumor, lymphatic and liver metastasis tumor respectively. The circle and triangle represent the mutation state at the position 2897- and 1350-point site of mitochondria. Their colors represent the mutation status: blue, orange, and red represent wild-type, heterozygous, and homozygous mutations respectively. PT: primary tumors and R1-R3 represent different regions of primary tumor. **C** Heatmap showing selected mitochondrial mutations of patient #9. Orange represent heterozygous mutations and red represents homozygous mutations. Blue represent wild-type and gray represents read depth lower than 9. Cells from PTR1 and liver metastasis have a chrM:2897 heterozygous mutation. PTR2 has region-specific mutations on chrM:11380 and chrM:8534. The bars above the heatmap shows the CNV subclones and tissue origin of the cells. The full list of mitochondrial mutations can be found in supplementary table 5. PT: primary tumors and R1-R3 represent different regions of primary tumor. LyM: lymph node metastasis. LM: liver metastasis and LM1 and LM2 represent two separated liver metastatic tumors. **D** Heatmap showing the reginal distribution of somatic mutations in all samples from patient #9. In total, 136 somatic mutations that were shared by at least two samples were identified. Red represent mutant state and gray represent wild-type state (left panel). Phylogenetic tree of lesions of patient #9 based on somatic mutations calling by WES data and the pie charts reflect the proportion of different subclones in each region based on CNVs that inferred by scRNA-Seq data

**Fig. 5:**
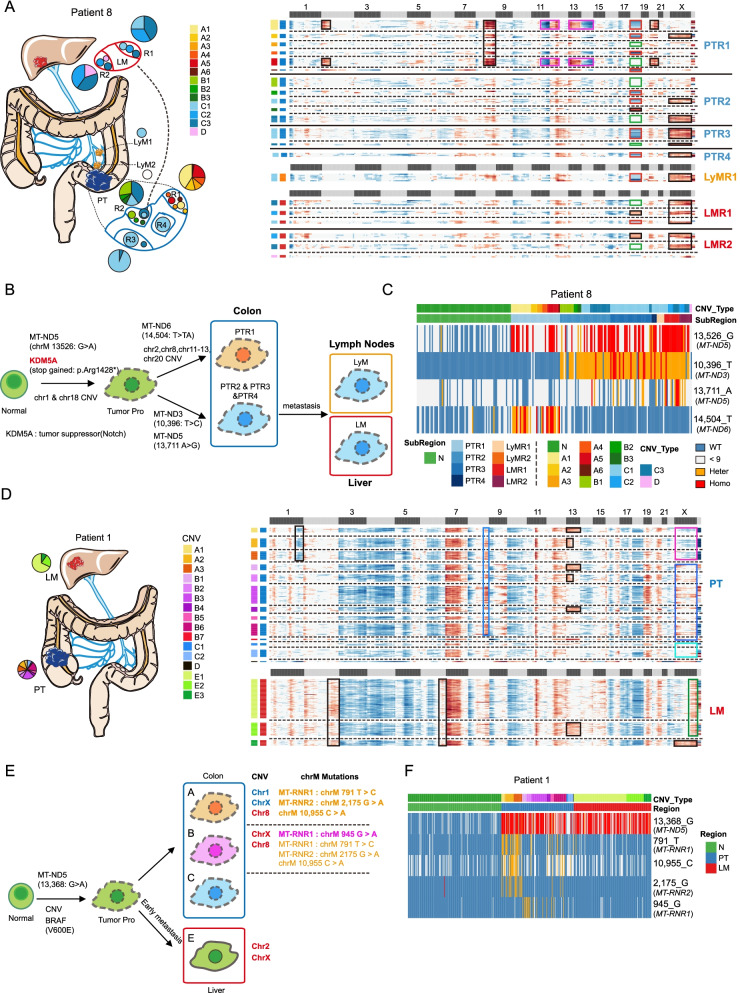
Possible presence of tumor precursor cells. **A** CNV pattern and sampling strategy of patient #8. N: adjacent normal tissues. PT: primary tumors and R1-R4 represent different regions of primary tumor. LyM: lymph node metastasis LyM1 and LyM2 represent two separated lymph node metastatic tumors. LM: liver metastasis. R1-R2 represents different regions of the liver metastatic tumors. The pie charts reflect the proportion of different subclones in each region. Copy number gain and copy number loss were indicated with red and blue respectively. Different tissues are divided by bold black solid lines, and different clones within the same tissue block are divided by black dashed lines. The squares above the heatmap represent different chromosomes, black squares represent odd-numbered chromosomes and chromosome X, and light gray squares represent even-numbered chromosomes and chromosome Y. The squares on the left of the heatmap represent different subclones, and the same color represents the same clone. The most obvious copy number differences between different clones were highlighted by different colored boxes. **B** The diagram showing the tumor metastasis path of patient #8. CNVs, somatic mutations and mitochondrial mutations are also shown in the diagram. The color of box represents different areas; blue, orange, and red box represent the primary tumor, lymphatic, and liver metastasis tumor respectively. Most of primary tumor cells have homozygous mutation at MT-ND5:13,536G>A mutation; thus, we speculated that there may be a group of tumor progenitor cells that we did not capture, which have *MT-ND5*(13,536G>A) mutation. With the development of the tumor, this group of tumor progenitor cells have additional mutations at *MT*-*ND6*(14,504 T>TA) and *MT-ND3*(10,396T>C) respectively, thus forming two clones (PTR1 A clones and PTR2-PTR4 B&C clones). Then the clones with *MT*-*ND3*(10,396T>C) mutation were further metastasized to the liver. **C** Heatmap showing selected mitochondrial mutations of patient #8. Almost all tumor cells have a chrM:13,526 homozygous mutation. Cells from the PTR1 (primary tumor region 1) have a chrM:14,504 mutation, and cells from PTR2-PTR4 and lymph node metastasis have a chrM:10,396 mutation. Blue represents wild-type and gray represents read depth lower than 9. Orange represent heterozygous mutation and red represent homozygous mutation. The bars above the heatmap shows the CNV subclones and tissue origin of the cells. **D** CNV pattern and sampling strategy of patient #1. PT: primary tumors. LM: liver metastasis. Copy number gain and copy number loss were indicated with red and blue respectively. Different tissues were divided by bold black solid lines, and different clones within the same tissue block were divided by black dashed lines. The squares above the heatmap represent different chromosomes, black squares represent odd-numbered chromosomes and chromosome X, and light gray squares represent even-numbered chromosomes and chromosome Y. The squares on the left of the heatmap represent different subclones, and the same color represents the same clone. The most obvious copy number differences between different clones are highlighted by different colored boxes. The black square in chromosome 1 shows A1–A3 subclone-specific CNV. The black squares in chromosome 2 and chromosome 6 show additional CNVs of LM (E1–E3 clone-specific clones) compared with PT. **E** The diagram showing the tumor metastasis path of patient #1. CNVs, somatic mutations and mitochondrial mutations are also shown in the diagram. **F** Heatmap showing selected mitochondrial mutations of patient #1. All tumor cells have homozygous mutation at 13,368 point site. N: adjacent normal tissue. PT: primary tumor. LM: liver metastasis

**Fig. 6:**
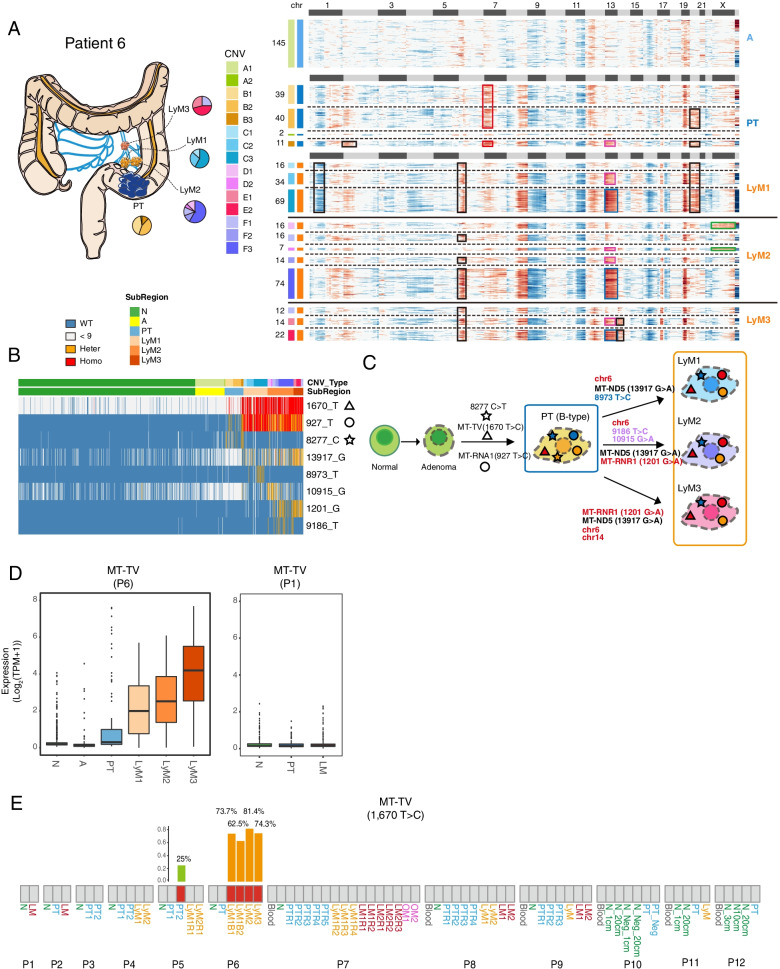
Integrated analyses of the associations between mitochondrial mutations and mitochondrial gene expression profiles. **A** CNV pattern and sampling strategy of patient #6. Adenomas have no obvious CNVs and different subclones with distinct CNV patterns are found in primary tumors. Lymph node metastasis has different CNV patterns with primary tumors. A: adenoma. PT: primary tumors. LyM: lymph node metastasis; LyM1, LyM2, and LyM3 represent three separated lymph node metastatic tumors. The pie charts reflect the proportion of different subclones in each region. Copy number gain and copy number loss were indicated with red and blue respectively. Different tissues were divided by bold black solid lines, and different clones within the same tissue block were divided by black dashed lines. The squares above the heatmap represent different chromosomes, black squares represent odd-numbered chromosomes and chromosome X, and light gray squares represent even-numbered chromosomes and chromosome Y. The squares on the left of the heatmap represent different subclones, and the same color represents the same clone. The most obvious copy number differences between different clones were highlighted by different colored boxes. **B** Heatmap showing selected mitochondrial mutations of patient #6. Blue, orange, red, and gray represent wild-type, heterozygous, and homozygous mutations and undefined (read depth < 9X) respectively. The triangle, circle and star represent the mutation state at the position 1,670-, 927-, and 8277-point site of mitochondria. **C** The diagram showing the tumor metastasis path of patient #6. The triangle, circle, and star represent the mutation state at the position 1,670-, 927-, and 8277-point site of mitochondria. Their colors represent the mutation status: blue, orange, red, and gray represent wild-type, heterozygous, and homozygous mutations respectively. **D** The boxplot showing the relative expression levels of MT-TV for patient #6 and patient #1. Other patients without MT-TV mutations have a similar pattern as patient #1 (data not shown). N: adjacent normal tissue. A: adenoma. PT: primary tumor. LyM: lymph node metastasis and LyM1-LyM3 represent three separated lymph node metastatic tumors. **E** The mutation state of mitochondrial gene *MT-TV* (site: 1,670) that is defined by whole exome sequencing data. Gray rectangle represents the wild-type, and the red rectangle represents the mutant type. The bar plot shows the allele frequency of mutations at this site. N: adjacent normal tissue. A: adenoma. PT: primary tumor. LyM: lymph node metastasis. LM: liver metastasis. For patients #10–#12, cells in the adjacent normal tissues were enriched by MACs or FACs; N represents EPCAM-positive cells and N_Neg represents EPCAM-negative cells. 1 cm, 3 cm, 10 cm, and 20 cm represent the distance to the edges of the tumor

We used single-cell RNA-Seq data to infer the CNVs of epithelial cells at single-cell resolution, and the CNV patterns were verified by WGS of the corresponding tumor tissues (Additional file 2: Fig. S6A). Except for patient #5 whose tumor was classified as an MSI tumor, tumor cells from all other patients showed distinct CNV patterns (Figs. 4, 5, and 6 and Additional file 2: Fig. S6-S8). In each patient, multiple CNV patterns were revealed. So we explored the evolutionary relationships between PTs, LyMs, and distant metastases based on their CNV patterns. Recently, it was reported that mitochondrial mutations enable study of clonal architecture using single-cell RNA-Seq data [38]. Therefore, we also used mitochondrial mutations to infer tumor cell lineages at single-cell resolutions.

In all the patients we analyzed (including patient #5 that classified as having an MSI-H tumor), we identified tumor-specific mitochondrial mutations and these mitochondrial mutations can serve as characteristic markers of the tumor cells for lineage tracing and identification of tumor cells without obvious CNVs. For example, since patient #5 has an MSI tumor, tumor cells and normal epithelial cells cannot be distinguished by CNV patterns (Additional file 2: Fig. S6A). However, all tumor cells of patient #5 had mutations in *MT-CO1* (chrM:7352), while normal epithelial cells from the same patient did not have this mutation (Additional file 2: Fig. S9A-B). In patient #3, a small number of cells without clear CNVs (D-type) from tumor tissues clustered with adjacent normal cells based on transcriptome data, and these cells did not have mutations in *MT-RNR2* (chrM:3004), which was shared by tumor cells with clear CNVs (Additional file 2: Fig. S6B-D).

Notably, lineage inference by CNVs and mitochondrial mutations showed high consistency with each other. Combining mitochondrial mutations and CNVs in the same individual cells can not only make the tumor clonal relations reconstructed more accurately and robustly but also be used to unambiguously distinguish the normal epithelial cells from tumor cells without obvious CNVs in tumor regions (Additional file 2: Fig. S6C).

### Mitochondrial mutations and CNVs at single-cell levels both reflect distinct origins of lymph node and different distal metastases in the same patients

Distant metastases are prevailingly believed to originate from lymph node metastases, but this model was challenged recently by constructing evolutionary relationship just based on 20–43 indels in polyguanine repeats [7]. Both CNV and mitochondrial mutation tracing revealed that the tumor epithelial cells from the lymph node and liver metastatic tumors of patient #9 had distinct origins (Fig. 4A–C). In patient #9, more than one metastasis sites existed, but tumor cells from different metastatic sites exhibited different CNV patterns and different mitochondrial mutations. Tumor cells from lymph node (LyM) showed similar patterns with the tumor cells in the center part of the primary tumor (PTR3), while tumor cells from liver metastasis showed patterns similar to the tumor cells located at the outer part of the primary tumor (PTR1 and PTR2) (Fig. 4A–C). Tumor cells from primary tumor region 3 (PTR3) and lymph node metastasis (LyM) had amplification on chr21 but did not have mutation on *MT-RNR2* (chrM:2897). So we deduced that after PTR3 tumor cells migrated to lymph nodes, these cells further produced new mutations at chrM:11380 and chrM:8534. On the other hand, tumor cells of primary tumor region 1 (PTR1) and liver metastasis (LM1 and LM2) had similar CNV patterns and all of them had “heterozygous” mutations at *MT-RNR2*(chrM:2897). In addition, constructing tumor phylogenetic tree based on WES of bulk samples in the patient also exhibited similar lineage patterns, further verifying the conclusion (Fig. 4D).

This phenomenon was consistent with the result of tumor evolution tracing based on 20–43 indels in polyguanine repeats reported by Naxerova *et al*. [7]. However, here, we traced these tumor clones at single-cell resolution instead of at the bulk level. Notably, the result of both CNVs and point mutations support independent origins of cancer cells in lymph node and liver metastases in the same patient. Compared with hypermutable loci, we can see much more detailed and accurate changes in tumor evolution with mitochondrial mutations which has faster mutation rate (over 10- to 100-fold faster), higher copy number changes (100–1000s in a cell), single point mutation resolution, and more tracking sites available (16.6 kb). By using single-cell transcriptome data, we can not only trace the tumor’s clonal structure, but also combine mutation identification or clonal structure identification with phenotypic assay at transcriptome levels.

More importantly, patient #7 has two different distal metastases, liver metastases, and omentum metastases, but these two metastasis sites showed distinct origins (Additional file 2: Fig. S7). Notable, liver metastases may originate from lymph node metastases but omentum metastases may directly originate from the primary tumor. According to the CNV patterns, both lymph node and liver metastases have a gain copy number at chromosome 2, while omentum metastases showed opposite CNV patterns at chromosome 2 (Additional file 2: Fig. S7A).

### The potential existence of tumor precursor cells

Tumor precursors should exist and later develop into different subclones. For patient #8, two distinct subclones existed in the primary tumor and only C-type tumor cells located in primary tumor region 2 and 3 (PTR2 and PTR3) migrated to the lymph node and liver, and further developed into D-type tumor cells in the liver metastatic region (Fig. 5A–C). Tumor cells in primary tumor region 1 (PTR1) had additional CNVs on chromosomes 2, 9, 11, 12, 13, and 20. In addition, tumor cells of PTR1 and other regions had different mutations at *MT-ND6*(chrM:14504 - PTR1) and *MT-ND3*(chrM:10396 - PTR2-PTR3, LyM, LM), respectively. However, all the tumor cells had mutations at *MT-ND5*(chrM:13526), which indicates that tumor precursor cells may exist. The proposed tumor precursor cells first (chrM:13526) migrate to PTR1 (A-types) and PTR2-PTR4 (B-types and C-types) and then become two different subclones at early stage, and these two subclones generated new subclone-specific mutations later.

Besides patient #8, mitochondrial mutation of patient #1 also supported the presence of tumor precursor cells (Fig. 5D–F). Although there were several subclones with different CNV patterns in the primary tumor of patient #1, all tumor cells have mitochondrial mutations at position 13368. This suggests that there was a group of tumor precursor cells with chrM:13368 mutation, and during tumorigenesis, it accumulated different genetic mutations and resulted in distinct subclones. Notably, the chrM: 13,368 of essentially all tumor cells were homoplasmic (Fig. 5F). Since there were hundreds of copies of mitochondrial DNAs in an individual cell [39], it needs to take multiple cell divisions and selection to replace all wild-type mitochondria with mutant ones, which may take relatively long time. The homoplasmic mitochondrial mutation of 13,368 further support the existence of tumor precursor cells in this patient, and it also indicated that cells with mitochondrial mutation at 13,368 may have a growth advantage compared with those with wild-type mitochondrial DNAs.

### Subclones exist in normal epithelial cells of the MSI CRC patient

In all nine patients, we investigated mitochondrial mutation spectrums, only patient #5 who was classified as MSI-positive tumor has normal epithelial cell-specific mitochondrial mutations. Moreover, three subclones were identified in normal epithelial cells in patient #5, which were mainly distinguished by wild-type, heteroplasmic, and homoplasmic positions like chrM:9576 (Additional file 2: Fig. S9A and S9B). This is consistent with several studies that have identified somatic mutations in normal epithelial cell at bulk levels [13–16, 38], and here we further verified it at single-cell levels using mitochondrial mutations. However, since we did not sequence other tissues of this individual, it is possible that the different mitochondrial mutations in normal and tumor tissue may be due to different developmental origins.

### Mutant mitochondrial DNA may have a higher expansion capacity

Interestingly, in patient #2, majority of the tumor epithelial cells in the primary tumor had a heteroplasmic mutation in *MT-ND3*(chrM:10192), but at liver metastasis (LM) region, almost all cells had homoplasmic mutations at *MT-ND3*(chrM:10192) (Additional file 2: Fig. S8A-B). This may suggest that tumor cells with homoplasmic mutant of mitochondria are more likely to metastasize or that mutated mitochondrial DNAs may expand during tumor progression and eventually completely replace wild-type mitochondrial DNAs. However, we observed this phenomenon in just one patient, and more patients need to be analyzed to further verify this interesting phenomenon in the future. In addition, mitochondrial mutations common to different subclonal tumor cells in different tumor regions are basically homoplasmic mutations (*MT-ND5*: 13368 G>T in patient #1, *MT-CO1*: 7352 T>C in patient #5, *MT-ND1*: 4049 G>A in patient #7, *MT-ND5*: 13526 C>A in patient #8), while most of the mutations shared by some subclonal cells are heteroplasmic mutations (Fig. 5C, F and Additional file 2: Fig. S7, S9A). This indicates that these homoplasmic mitochondrial mutations were generated much earlier and may go through selection before it became homoplasmic and completely replaced wild-type mitochondrial DNAs in cancer cells in these patients. However, in addition to selection, genetic drift and stochastic bottleneck effects may also contribute to the homoplasmic phenomena. For example, the high rate of cell turnover and asymmetric segregation of mtDNAs during cell divisions could also lead to homoplasmic mtDNA variants in cells. The main difference between selection and genetic drift is whether changes in allele frequencies are random. Unlike selection, genetic drift does not depend on an allele’s beneficial or harmful effects. Instead, it changes allele frequencies purely by chance. Since most of our samples were from patients before treatment, cells basically do not experience large-scale cell death, so the possibility of homoplasmic due to selection is higher, but the possibility of genetic drift cannot be fully ruled out. More samples and experiments are needed in the future to further judge this conclusion.

### MT-TV upregulation in tumor cells may be attributed to mutation of the gene

Although many studies have revealed the genomic and transcriptomic features of cancer cells, how genotypic and phenotypic features are interconnected is still poorly understood. To this end, we systematically explored the association between gene expression profiles and point mutations at a single-cell resolution in mitochondrial and nuclear DNAs, respectively.

Unexpectedly, our data revealed that mitochondrial mutations may regulate mitochondrial gene expression. In patient #6, tumor cells had mutations at *MT-TV* (chrM:1670) and *MT-RNR1*(chrM:927) sites (Fig. 6A–D). However, *MT-TV* (mitochondrially encoded tRNA valine) genes were hardly detected in the normal epithelial cells from adjacent normal tissues, and even if they were expressed in some of these cells, the expressed alleles were wild-type ones (Fig. 6B). Then, we investigated the expression level of *MT-TV* genes in tumor cells, and only tumor epithelial cells with *MT-TV* mutation (chrM:1670) showed increased expression of *MT-TV* (Fig. 6D). In addition, we also explored the expression levels of *MT-TV* in other patients who did not have *MT-TV* mutations and found that the tumor epithelial cells from all other patients analyzed express *MT-TV* at very low levels (Fig. 6D). We also explored the *MT-TV* expression by using the data of Genotype-Tissue Expression (GTEx) Project database which collected RNA expression of normal tissues. Only cells from the brain showed very low levels of *MT-TV *expression, and essentially all other normal cells from other tissues do not express *MT-TV* ﻿(https://genome.ucsc.edu/cgi-bin/hgGene?db=hg38&hgg_gene=MT-TV) [40]. Since we called mitochondrial mutations using single-cell RNA-Seq data, if the gene is not expressed, we cannot judge whether the corresponding mitochondrial DNA region has a mutation or not. To circumvent the limitation of SNP calling with RNA-seq data, we performed whole exome sequencing on the same sample at bulk level to determine whether the sample has specific mitochondrial mutations at the DNA level (Fig. 6E). According to the WES data, *MT-TV* mutation was detected in all lymphatic metastases of patient #6, and the mutation frequency was over 62.5%, which means that almost all tumor cells have this mutation considering that bulk tumor samples contain significant proportion of nonepithelial cells such as fibroblasts and immune cells. We also detected this mutation in one primary tumor (PT2) of patient #5, but the mutation frequency is relatively low, only 25%. Through single-cell RNA-seq data, we found that all cells we picked in PT2 of patient #5 have wild-type mitochondrial DNAs (Additional file 1: Table S4). These data indicated that some specific mitochondrial mutations may also contribute to tumorigenesis of CRC and affect gene expression patterns of cancer cells.

### Metastasis of TP53^mut^ tumor cells benefits from EKC/KEOPS complex overexpression

**Fig. 7:**
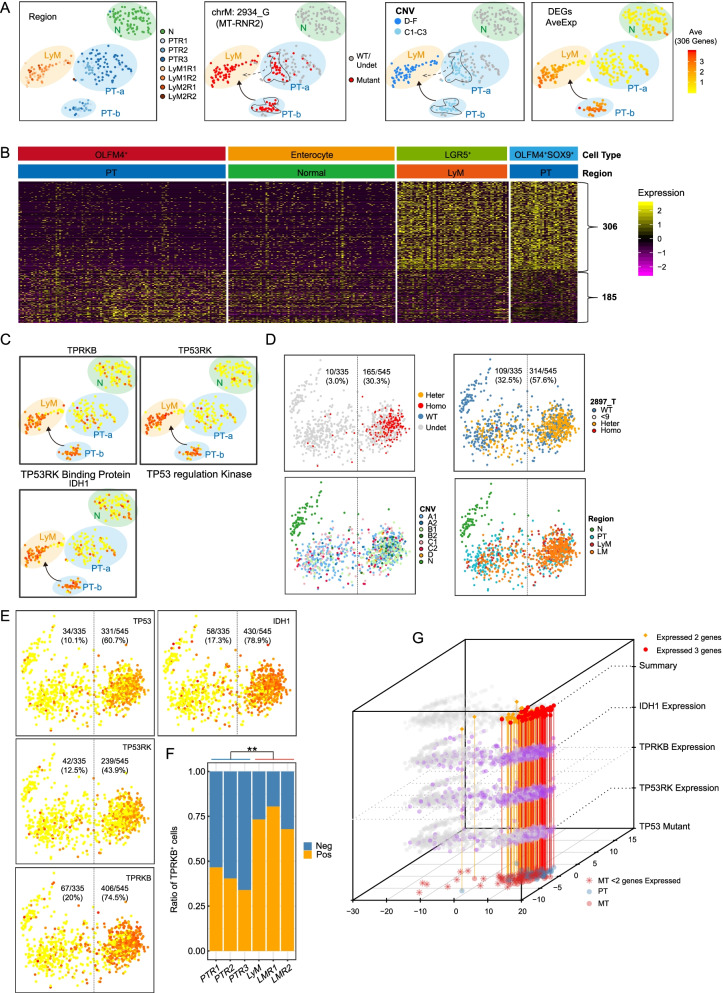
Integrated analyses of the associations between TP53 mutation and global gene expression profiles. **A** The tSNE map of patient #4, which has a TP53 frame-shift mutation. Cells were colored according to tissue origin, mitochondrial mutation, merge CNV types, and average expression levels (log_2_(TPM+1)) of 306 primary tumor OLFM4^+^SOX9^+^ group specific genes, which are shown in **B**. N: adjacent normal tissue. PT: primary tumor. LyM: lymph node metastasis. LM: liver metastasis. Cells from primary tumor were clustered into two groups, PT-a and PT-b. Associated with Fig. S10A. **B** Heatmap shows the expression pattern of DEGs between OLFM4^+^ SOX9^+^, and OLFM4^+^ in all four cell groups of patient #4 C. The expression levels of *IDH1*, *TPRKB* (TP53RK-binding protein), and *TP53RK* (TP53 regulation kinase) were projected on tSNE maps. Colors from yellow to red represent expression levels from low to high. **D** PCA clustering of patient #9 epithelial cells. Cells were colored by TP53 Sanger results, CNV type, cell origin, and mitochondrial mutation (chrM:2897). N: adjacent normal tissue. PT: primary tumor. LyM: lymph node metastasis. LM: liver metastasis. **E** The expression levels of *TP53*, *TPRKB*, *TP53PK*, and *IDH1* were projected to the PCA maps of patient #9 epithelial cells. TP53^High^ group means tumor epithelial cells that expressed TP53 gene according to the single-cell RNA sequencing data, while TP53^Low^ group represent tumor epithelial cells that did not expressed *TP53*. **F** The ratio of tumor epithelial cells that expressed TPRKB genes for each region. Metastatic tumor regions had more cells expressing *TPRKB*. ** represents *p*-value < 0.05. N: adjacent normal tissue. PT: primary tumor. LyM: lymph node metastasis. LM: liver metastasis. Neg represent *TPRKB* negative cells and Pos represent *TPRKB* positive cells. **G** The three dimensions plot shows the relationship between *IDH1*, *TPRKB*, *TP53RK* expression with *TP53* mutations. The XY plane shows the PCA result of Patient #9. The three layers of *IDH1*, *TPRKB*, and *TP53RK* show the expression levels of these three genes on PCA map. The summary layer shows cells with *TP53* mutations (according to Sanger sequencing). Red represents cells with *TP53* mutation and expressed all three genes (*IDH1*, *TPRKB* and *TP53RK*) simultaneously, while yellow represent cells with *TP53* mutation and expressed two of these genes. The *TP53* Mutant layer shows the tissue origin of cells with TP53 mutations. Red represents cells collected from metastatic tumor and blue represents cells collected from primary tumor

The tumor suppressor gene TP53 is one of the most frequently mutated genes in human tumor epithelial cells. In our cohort, five patients had *TP53* mutations (patients #2, #4, #6, #9, and #10), and two of these patients (patients #4 and #9) had WES and scRNA-seq data for both primary tumor and metastatic tumors (Additional file 1: Table S9). Integrated analysis of *TP53* mutation, lineage inferences, and single-cell expression profiles revealed that for the same subclone of tumor cells that metastasized to the liver, some cancer cells in the primary tumor and essentially all of the cancer cells in the metastasized tumor highly expressed EKC/KEOPS complex. This indicates that the cancer cells within this subclone that specifically upregulated EKC/KEOPS is more likely to metastasize to the liver successfully compared with those cancer cells that did not express EKC/KEOPS (Fig. 7 and Additional file 1: Table S5-S6).

In patient #4, cancer cells from primary tumors (PT1 and PT2) and lymph node metastasis (LyM1 and LyM2) all had a *TP53* frame-shift insertion (chr17:7578502 A>ACAGGG). Based on mitochondrial mutations and CNVs, C-type tumor cells successfully metastasized to the lymph nodes (Additional file 2: Fig. S8C-D). However, according to the *t*-distributed stochastic neighbor embedding (tSNE) clustering based on the gene expression matrix, the C-type tumor cells separated into two subgroups (PT-a and PT-b) (Fig. 7A and Additional file 2: Fig. S10A). After applying the DEG analysis of these two groups of PT cells, the expression patterns of the DEGs in all epithelial cells of patient #4 were shown (Fig. 7A,B). LyM tumor cells (LGR5^+^ cells) also highly expressed signature genes of the PT-b cluster (Fig. 7A,B and Additional file 2: Fig. S10B). In details, both lymph node metastatic tumor cells and the primary tumor *OLFM4*^*+*^*SOX9*^*+*^ C-type cells highly expressed intestinal stem cell markers (*SOX9* and *LGR5*), *TP53RK* (*TP53* regulating kinase), and *TPRKB* (*TP53RK* binding protein) (Fig. 7C and Additional file 2: Fig. S10C). Both *TP53RK* and *TPRKB* are components of the evolutionarily conserved EKC/KEOPS complex (Endopeptidase-like Kinase Chromatin-associated protein complex/Kinase putative Endopeptidase and Other Proteins of Small size protein complex) that are required for the essential N6-theonylcarbamoyladenosine (t6A) modification of all ANN-codon recognizing tRNAs [41]. Based on the result of CNVs and mitochondrial mutations, we showed that lymph node metastasis originate from C-type tumor cells in the primary tumor (Additional file 2: Fig. S8C-D). And now based on gene expression patterns, it further indicated that lymph node metastatic tumor cells are more likely originated from *OLFM4*^*+*^*SOX9*^*+*^ C-type cells (PT-b), but not from *OLFM4*^*+*^*SOX9*^*-*^ C-type cells (PT-a) in the primary tumor (Fig. 7A–C and Additional file 2: Fig. S10A-C). So even though both groups of C-type cells have similar genomic mutation patterns, only cells that highly expressed *TP53RK* and *TPRKB* successfully metastasized to lymph node, which indicated that EKC/KEOPS complex may be beneficial for tumor metastasis (Fig. 7A, C).

In patient #9 who had missense mutations at *TP53*, we also observed that *TP53RK/ TPRKB*-positive cells were more likely to metastasize to distal organs (Fig. 7D–G). Unlike those in patient #4, the tumor cells in patient #9 clustered into two groups, *TP53*^*High*^ group and *TP53*^*Low*^ group. For tumor cells in which we detected *TP53* missense mutations with single-cell targeted cDNA Sanger sequencing, most of them were homozygous mutations and belonged to the *TP53*^*High*^ group (Figs. 7D and 6E and Additional file 1: Table S6). Moreover, a previous study verified that TP53-deficient cells were dependent on TPRKB through knockdown of *TPRKB* in TP53-deffficient and TP53 wild-type cell lines. They found that after knockdown of *TPRKB*, the growth of *TP53*-deficient cells was significantly inhibited but knockdown of *TPRKB* in *TP53* wild-type cells had minimal influences [42]. Here, through integrated analysis of CNVs, mitochondrial mutations, and gene expression patterns, we showed that EKC/KEOPS complex might play important role for metastasis of TP53-deficient tumor cells in CRC patients in vivo (Fig. 4A–C).

### BRAF^V600E^ mutation has little effect on global gene expression patterns

Approximately 8–15% of CRC tumors harbor BRAF mutations. Despite several BRAF inhibitors have emerged, unfortunately unlike other cancers, BRAF mutant CRC patients showed inefficient responses to these BRAF inhibitors [43]. Thus, we explored the relationship between the BRAF^V600E^ mutation and transcriptomic patterns (Additional file 2: Fig. S10D-G).

According to the clinical information and WES data, patient #1 harbored the *BRAF*^*V600E*^ mutation. Combined with single-cell targeted cDNA Sanger sequencing (Additional file 1: Table S6), we separated the tumor epithelial cells of patient #1 into two groups, *BRAF*^*WT*^ and *BRAF*^*V600E*^. Through PCA analysis, all epithelial cells of patient #1 clustered into three groups based on their cell origins. The first PCA component separated tumor cells from normal epithelial cells, while the second PCA component separated primary tumor cells from liver metastatic tumor cells (Additional file 2: Fig. S10D). However, regardless of *BRAF* mutation, cells clustered together by tumor regions, which may imply that the single *BRAF*^*V600E*^ mutation may not cause dramatic changes in global gene expression patterns of cancer cells (Additional file 2: Fig. S10D).

According to clonal inference by mitochondrial mutations and CNVs, liver metastases of patient #1 may derive from another subclone in the primary tumor region that we may not capture (Fig. 5D–F). To avoid the impact of subclones, we performed the DEG analysis between *BRAF*^*V600E*^ and *BRAF*^*WT*^ epithelial cells from the PT and LM regions respectively (Additional file 2: Fig. S10E, S10F and Additional file 1: Table S7). The DEGs that were identified in the LM region were much more abundant than those in the PT region. *BRAF*^*WT*^ tumor cells of the PT region highly expressed *BMP7*, whose elevated expression was known to be correlated with tumor invasion, metastasis, recurrence, and cancer-related death (Additional file 1: Fig. S10F) [44]. Tumor cells with *BRAF*^*V600E*^ mutation from PT and LM all highly expressed *VPS13B*, a gene that may function in vesicle-mediated transport, and *L3MBTL2*, a gene that may inhibit cell divisions. In our cohort, there was only one patient having BRAF^V600E^ mutation. Therefore, we combined TCGA-COAD data to check if the specifically upregulated genes in BRAF^V600E^ cells were widely applicable. We divided TCGA-COAD into two groups: BRAF^V600E^ and BRAF^600WT^, and we found that the expression of *L3MBTL2* in BRAF mutant tumors was significantly higher than that in BRAF wild-type tumors (Additional file 2: Fig. S10G). As for the other gene *VPS13B*, there were no significant differences between the wild-type and BRAF mutant tumors of TCGA, which may be masked by the influences of other microenvironmental cells in the bulk tumor samples.

### KRAS mutation mainly affects actively dividing tumor cells

Approximately 40–50% of CRCs have a mutated *KRAS* oncogene, and the most common mutations of KRAS are in codons 12 and 13 [45]. According to the WES results, patient #9 had a *KRAS*^*G12V*^ mutation (Additional file 1: Table S8). Combined with single-cell targeted cDNA Sanger sequencing, we separated tumor epithelial cells of patient #9 into three groups, *KRAS*^*WT*^, heterozygous *KRAS*^*G12V*^, and homozygous *KRAS*^*G12V*^ (Additional file 2: Fig. S10H and Additional file 1: Table S6). Based on gene expression patterns, cells were clustered into two groups, actively dividing cells (*MKI67*^*+*^) and quiescent cells (*MKI67*^*−*^) (Additional file 2: Fig. S10I). To avoid the influence of different tumor regions or cell cycle phases, we performed DEG analysis between *KRAS*^*WT*^ and *KRAS*^*G12V*^ cells separately for each tumor site (Additional file 2: Fig. S10J-K and Additional file 1: Table S8). The actively dividing tumor cells have more DEGs between *KRAS*^*WT*^ and *KRAS*^*G12V*^, no matter when these cells are from the primary tumor or metastatic tumor (Additional file 1: Table S8). This indicates that mutations in *KRAS* may have a greater impact on actively dividing tumor cells than on quiescent tumor cells.

In summary, through precise comparisons of different subclones of cancer cells in vivo from the same patients based on point mutations, CNVs, and transcriptomic characteristics all at single-cell resolutions, our data clearly offers mechanistic insights for the potential functional involvement of driver mutations (such as *TP53*, *BRAF*, and *KRAS*) in CRC patients in vivo.

## Discussion

Here, we report a comprehensive transcriptomic analysis of eleven advanced CRCs at single-cell levels. Multi-region sampling was performed for the tumor samples that we collected from most of the patients we analyzed. Through single-cell RNA-Seq analysis, we were able to investigate the dynamic changes in cell-type compositions during tumor progression. The identified DEGs and tumor-specifically activated signaling pathways could serve as clinical biomarkers and potential therapy targets, which may be used to achieve better CRC diagnoses in the future. Through the identification of global CNV patterns and mitochondrial mutations from the single-cell RNA-seq data among CRCs, different subclones were identified in a single tumor region and the interconnection of genotypic and phenotypic features was unveiled in CRC at a single-cell resolution. Taken together, our findings provide a rich resource for understanding the heterogeneities of the metastatic CRCs at different omics layers.

One of our key findings was a drastic increase in the proportion of intestinal stem/progenitor-like cells and Paneth cells in tumor epithelial cells, accompanied by a sharp decrease in highly differentiated mature intestinal epithelial cells, such as enterocytes and goblet cells. Through establishing patient-derived cancer organoids, we found that stem cell-like cells (*SOX9*^*+*^ and *MKI67*^*+*^) may be the main component of tumor epithelial cells in vivo and that these cells can be self-renewal and further differentiate into other mature cell types. However, since it is hard to enrich *SOX9/MKI67*-positive living cells through FACS because of the nuclear localization of these marker genes, we did not perform experiments such as extreme limiting dilution assay to further verify the self-renewal and differentiation ability of these stem cell-like cells. Therefore, we cannot fully exclude the possibility that these phenomena may be due to the plasticity of the cancer cells. Moreover, the PPAR signaling pathway was prevalently and aberrantly activated in tumor cells and inhibition of this signaling pathway through PPARγ inhibitor could both dramatically suppress the growth and accelerate the apoptosis of the tumor epithelial cells. Although these have been verified using in vitro cultured organoids, in the future in vivo experiments are still needed for further verification of the function of PPAR signaling pathway. In addition, we have observed that when a specific concentration of inhibitors is used to inhibit both WNT and PPAR signaling pathways, the tumor killing effect is much greater than that of only inhibiting a single pathway alone. Although we have described the changes in the transcriptome after dual-inhibitor FH535 treatment through RNA-Seq, the study of underlying molecular mechanisms still requires further explorations, such as knock-out mouse model experiments.

Our data also provide insights into the mechanisms of CRC metastasis through clonal lineage tracking with both CNV patterns and mitochondrial mutations at single-cell resolution. In general, more than one subclones coexisted in a single primary tumor tissue, and some of these subclones have higher capacity to metastasize to lymph nodes or distant organs (such as B clones in patient #8 and C clones in patient #4). For some patients, it showed that metastatic tumors acquired additional properties compared to primary tumors (such as patients #2, #4, and #6). The current data cannot explain whether this phenomenon is caused by the acquisition of characteristics before metastasis or the selection at metastatic sites. Furthermore, our data provide evidences that different metastatic sites were invaded by different subclones and different metastasis occurred independently, which may not agree with traditional ideas that distant organ metastasis was seeded through lymph nodes (such as patient #7 and patient #9). These findings can shed light on the understanding of CRC tumor progression.

Notably, our data allowed us to explore the relationships between gene expression and genome variations, such as CNVs and point mutations in either mitochondria DNAs or nuclei DNAs at single-cell resolution. Although there are only a small number of genes in mitochondrial genome compared with nuclear genome, these mitochondrial mutations can still cause a variety of human genetic diseases and exhibits maternal inheritance characteristics as expected. In patient #6, we found that mutations in the *MT-TV* genes of tumor cells may result in increased expression of *MT-TV* itself. However, since we just observed this phenomenon in just one patient, if it is prevalent still needs to be tested in a larger number of cases. In addition, the function of the *MT-TV* mutation has not been directly explored here, thus more functional experiments are needed to verify it in the future. To study the function of mitochondrial genes and the pathogenic mechanisms of mitochondrial genetic diseases, mitochondrial targeted high-efficiency gene editing tools are necessary. Recently, scientists had finally made precise gene editing to mitochondrial DNAs for the first time which makes it possible to precisely edit mitochondrial genome [46]. In addition, in vitro cultured organoid system can also be used to explore the mitochondrial mutations which may be achieved by importing mutated mitochondria from tumor organoids into normal organoids. In total, we performed single-cell targeted cDNA Sanger sequencing of two critical oncogenes (*KRAS* and *BRAF*) and two important tumor suppressor genes (*TP53* and *APC*) in patient #1 (*BRAF*) and patient #9 (*KRAS*, *TP53*, and *APC*). Integrated analysis of point mutations and RNA expression indicated that *TPRKB* and *TP53RK* expressed *TP53*-mutated tumor cells were more likely to metastasize compared with tumor cells that did not express *TPRKB* and *TP53RK*, which was consistent with previous report [42]. However, further experiments were still needed to explore the underlying molecular mechanisms between EKC/KEOPS and metastasis. But here, we firstly verified it in vivo, which can completely eliminate confounding differences in individual genetic background or deviations caused by in vitro culture. Intriguingly, more DEGs between *KRAS*^*G12V*^ and *KRAS*^*WT*^ were identified for actively dividing tumor cells, which suggested that KRAS mutations may mainly affected tumor cells that were actively dividing. Similarly, this conclusion still needs more data support and further functional experiment verification, such as targeted knock-in of specific mutation sites. In addition, we found that a single point mutation of *BRAF*^*V600E*^ may not result in dramatic changes in tumor cells’ global gene expression patterns. More importantly, we found that *BRAF*^*V600E*^ tumor cells highly expressed *L3MBTL2* whereas *BRAF*^*WT*^ tumor cells did not, which is further verified in TCGA-COAD database. To our best knowledge, this is the first time that targeted gene mutation and RNA expression were simultaneously analyzed in the same individual tumor cell in vivo from the colorectal cancer patients. Due to the small sample size, our conclusions may be not widely applicable and need further verifications in larger cohort, but our data still provide novel insights into how driver mutations interfere with the transcriptomic state of a tumor cell. Larger dataset and further genetic experiments in the future can provide additional information. Limitations of our study include relatively small number of patients analyzed and the lack of in vivo functional validations. But our analyses dissect the genomic and transcriptomic heterogeneities in CRCs in an integrated manner. We also provide essential information about metastatic mechanisms, potential novel markers, and potential therapeutic targets for CRC diagnosis and therapy. Our high-precision single-cell RNA-Seq dataset of matched adjacent normal tissues, primary tumors, and metastases from CRCs provides a rich resource for further studies of CRCs.

## Conclusions

Our data also provide insights into the mechanisms of CRC metastasis. In general, more than one subclone coexisted in a single primary tumor tissue. And subclones harboring more severe CNVs and being located in the central region of PTs seemed to be more likely to metastasize to distal organs. This may be because the central region of the tumor is usually more hypoxic where immune cells have difficulty to infiltrate and survive [47]. Furthermore, our data provide higher resolution and more robust evidence of lineage tracing with mitochondrial point mutations and genomic CNVs to support that different metastatic sites of the same patient were invaded by different tumor subclones independently, which had previously been raised by construction of phylogenetic trees with several hypermutable loci [7, 48]. These findings can shed light on the molecular understanding of tumorigenesis process of CRCs.

## Supplementary Information

**Additional file 1: Table S1.** Basic information of sample cohort. N, adjacent normal tissue. PT, primary tumor. LyM, lymph node metastasis. LM, liver metastasis. OM, omentummetastasis. LN, adjacent normal tissue collected from liver. **Table S2.** DEGs between normal epithelial cells and tumor epithelial cells. Related to Fig. 3A **Table S3.** Cell viability of organoids and DEGs between control and FH534-treated organoids. Related to Fig. 2. **Table S4.** Mitochondrial mutations of each patient. Only the sites where mutations were detected in at least two cells were listed in the table. WT, wild type. Undet, reads than least than 9X. Heter, heterozygous mutation. Homo, homozygous. T and N represent tumor and normal. **Table S5.** DEGs between OLFM4^+^SOX9^-^ and OLFM4^+^SOX9^+^ tumor cells. Related to Fig. S10A. **Table S6.** Single-cell targeted cDNA Sanger sequencing result. The number inside the box represents the number of the cell barcode. Green, orange and red colors represent wild type, heterozygous mutant and homozygous mutant respectively. No color fill represents undetected. **Table S7.** DEGs between BRAF^V600E^ and BRAF^600WT^ tumor cells. Related to Fig. S10C-D. **Table S8.** DEGs between KRAS^WT^ and KRAS^mut^ tumor cells. Related to Fig. S10H-I. **Table S9.** Somatic mutations of samples. **Table S10.** Antibody information and sequences of primers.

**Additional file 2: Figure S1.** Global transcriptomic patterns of CRC and cell type composition changes during tumorigenesis. **Figure S2.** Transcriptomic differences between adjacent normal tissues and tumor tissues. **Figure S3.** Lineage tracing and cell-type specific markers’ expression patterns in several single organoid spheres. **Figure S4.** IHC and immunofluorescence staining verification and survival curve analysis of tumor-specific genes. **Figure S5.** Comparison with TCGA dataset and inhibition of PPAR signaling pathway on organoid system. **Figure S6.** CNV pattern verification by WGS and tumor lineage inferences of Patient #3 by single-cell RNA-Seq data. **Figure S7.** Tumor lineage inferences of Patient # 7 by mitochondrial mutations and CNVs. **Figure S8.** Tumor lineage inferences of Patient #2 and Patient #4 by mitochondrial mutations and CNVs. **Figure S9.** Normal epithelial cell-specific mitochondrial mutations existed in MSI-H tumor and experimental flowchart for single-cell targeted cDNA Sanger sequencing verification. **Figure S10.** The relationship between BRAF^V600E^, KRAS^G12V^ mutations and gene expression profiles.

## Data Availability

Our transcriptome data, whole genome, and whole exome data have been deposited in Genome Sequence Archive (GSA) under accession number HRA000183 (https://bigd.big.ac.cn/gsa-human/browse/HRA000183) [49]. The script of main steps can be found in the GitHub (https://github.com/WRui/Metastatic-Colorectal-Cancer and https://github.com/WRui/Post_Implantation/tree/master/scRNA_UMI )[25, 26].

## Abbreviations

CRC: Colorectal cancer
WES: Whole exome sequencing
WGS: Whole genome sequencing
CNVs: Copy number variations
MSI: Microsatellite instability
MMR: Mismatch repair
