# Supplementary material for: Single-cell genomic and transcriptomic landscapes of primary and metastatic colorectal cancer tumors
Source: Genome Med. 2022 Aug 16;14:93. doi: 10.1186/s13073-022-01093-z (PMC9380328; doi:10.1186/s13073-022-01093-z)
Supplement: Supplementary file 2 — Additional file 2: Figure S1. Global transcriptomic patterns of CRC and cell type composition changes during tumorigenesis. Figure S2. Transcriptomic differences between adjacent normal tissues and tumor tissues. Figure S3. Lineage tracing and cell-type specific markers’ expression patterns in several single organoid spheres. Figure S4. IHC and immunofluorescence staining verification and survival curve analysis of tumor-specific genes. Figure S5. Comparison with TCGA dataset and inhibition of PPAR signaling pathway on organoid system. Figure S6. CNV pattern verification by WGS and tumor lineage inferences of Patient #3 by single-cell RNA-Seq data. Figure S7. Tumor lineage inferences of Patient # 7 by mitochondrial mutations and CNVs. Figure S8. Tumor lineage inferences of Patient #2 and Patient #4 by mitochondrial mutations and CNVs. Figure S9. Normal epithelial cell-specific mitochondrial mutations existed in MSI-H tumor and experimental flowchart for single-cell targeted cDNA Sanger sequencing verification. Figure S10. The relationship between BRAFV600E, KRASG12V mutations and gene expression profiles. [file 13073_2022_1093_MOESM2_ESM.pdf]

Fig.S1

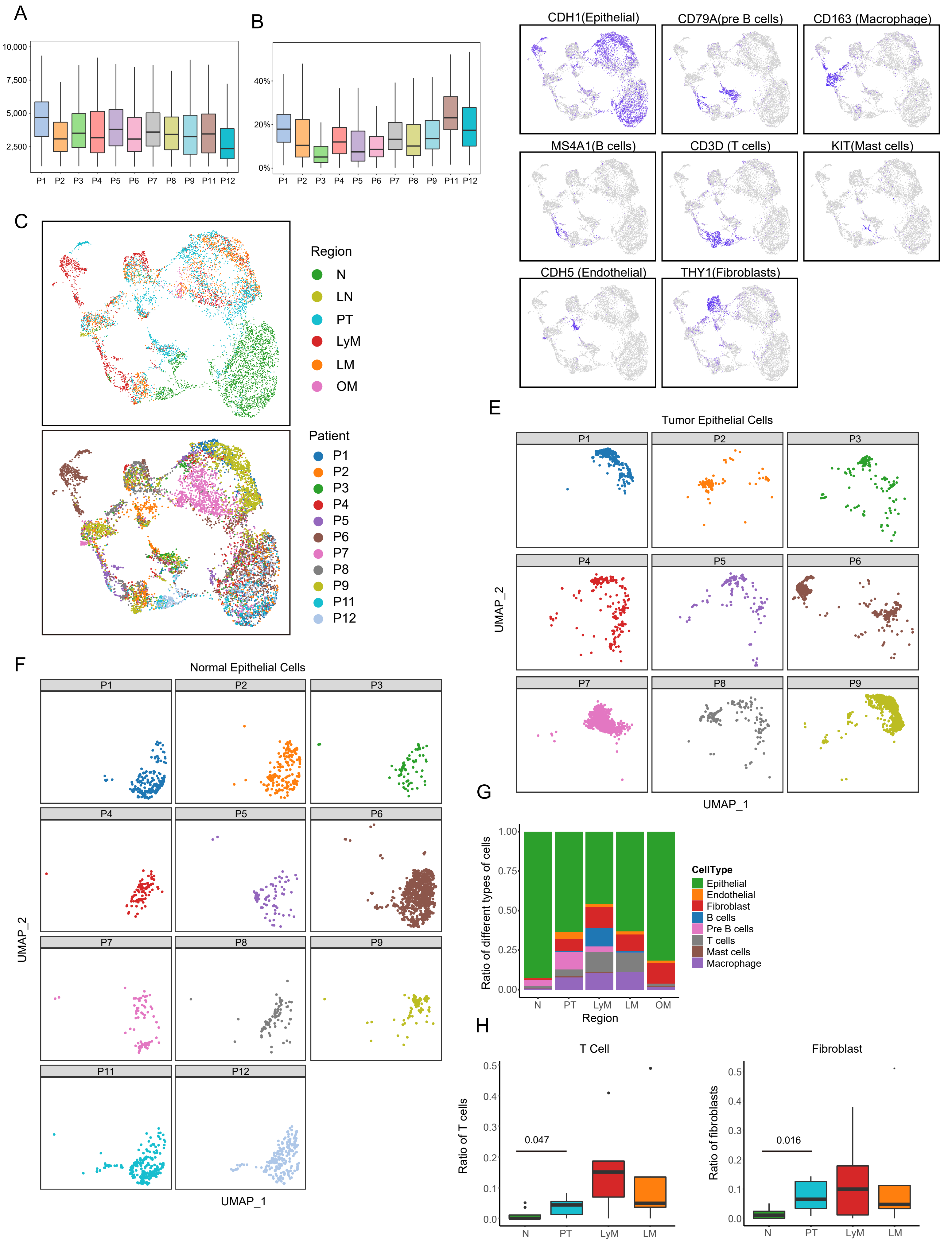

A
B
C
D
E
F
G
H
CDH1(Epithelial)
CD79A(pre B cells)
CD163 (Macrophage)
MS4A1(B cells)
CD3D (T cells)
KIT(Mast cells)
CDH5 (Endothelial)
THY1(Fibroblasts)
Region
N
LN
PT
LyM
LM
OM
Patient
P1
P2
P3
P4
P5
P6
P7
P8
P9
P11
P12
Tumor Epithelial Cells
Normal Epithelial Cells
UMAP_1
UMAP_2
Ratio of different types of cells
CellType
Epithelial
Endothelial
Fibroblast
B cells
Pre B cells
T cells
Mast cells
Macrophage
Region
T Cell
Fibroblast
Ratio of T cells
Ratio of fibroblasts
0.047
0.016

Fig. S1 Global transcriptomic patterns of CRC and cell type changes during tumorigenesis

A. Box plot showing the number of genes that detected in each individual cell for each patient. Different colors represent different patients.

B. Box plot showing the ratio of reads that mapped to the mitochondrial. The median ratio mitochondrial reads of all cells is 12.5%.

C. UMAP plot of all cells that passed the quality control. Cells were colored according to patient and tissue origin. N represents cells that collected from adjacent normal tissues. LN represents cells that collected from normal liver tissues. PT represents primary tumor. LyM, LM and OM represents cells that collected from lymph node, liver and omentum metastasis, respectively.

D. The expression levels of canonical cell-type specific marker genes were projected on the UMAP plot. Colors from gray to purple represent expression levels from low to high. *CDH1*, epithelial cell marker. *CD79A*, pan-B cell marker. *CD163*, macrophage marker. *MS4A1*, B cell marker. *CD3D*, T cell marker. *KIT*, mast cell marker. *CDH5*, endothelial cell marker. *THY1*, stromal cell marker.

E. UMAP of epithelial cells in tumor. Each point represents a single cell, different colors represent different patients. Tumor epithelial cells clustered according to the cell origin.

F. UMAP of epithelial cells in adjacent normal tissue. Each point represents a single cell, different colors represent different patients. Normal epithelial cells from different patients mixed well on the UMAP.

G. The bar plot shows the ratio of different cell types in different areas. Different colors represent different cell types. N, normal tissue; PT, primary tumor tissue; LyM, lymph node metastasis; LM, liver metastasis; OM, omentum metastasis.

H. The box plot shows the proportion of T cells and fibroblasts in different areas of each patient. N, normal tissue; PT, primary tumor tissue; LyM, lymph node metastasis; LM, liver metastasis.

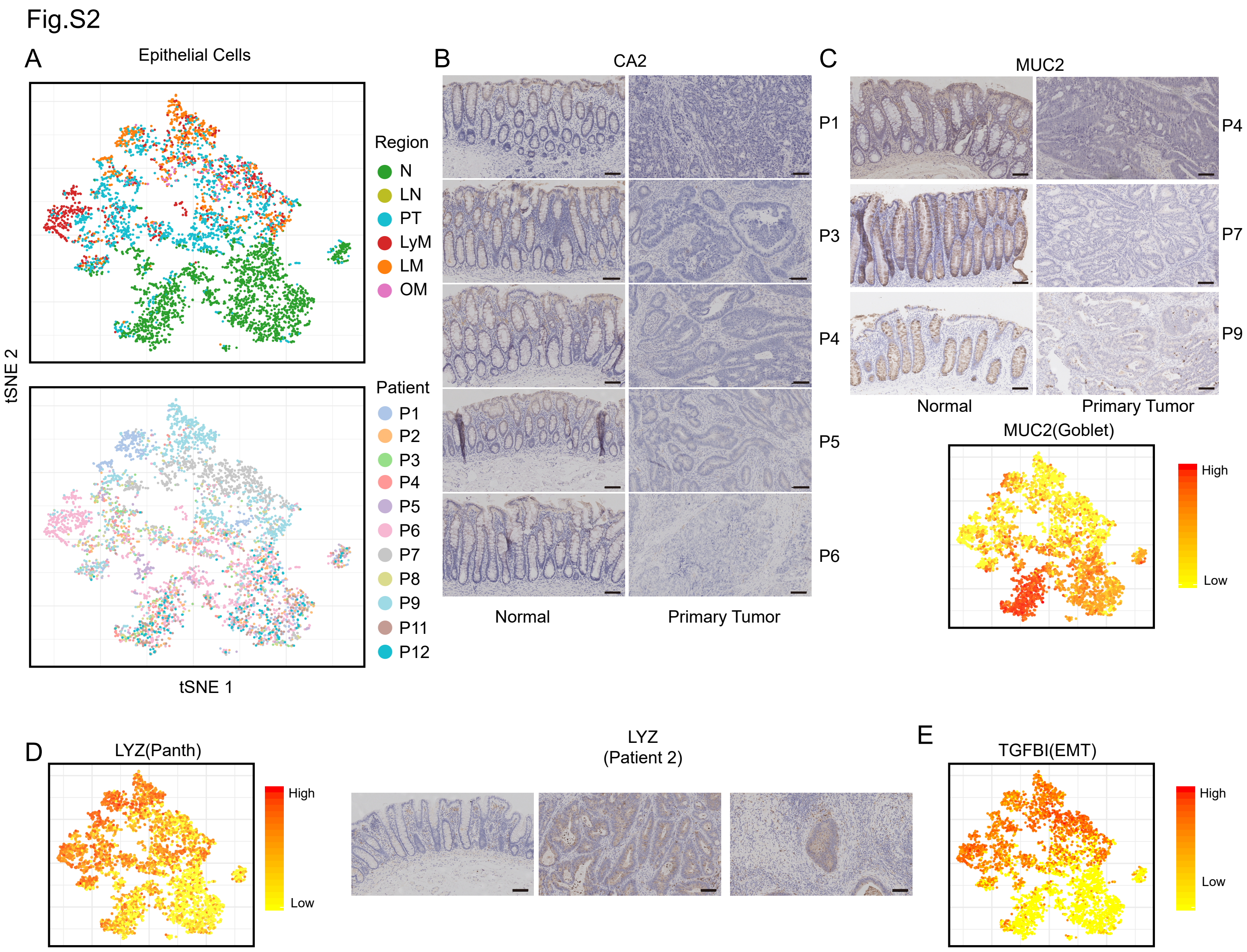
Fig.S2
A
Epithelial Cells
Region
N
LN
PT
LyM
LM
OM
Patient
P1
P2
P3
P4
P5
P6
P7
P8
P9
P11
P12
tSNE 2
tSNE 1
B
CA2
P1
P3
P4
P5
P6
Normal
Primary Tumor
C
MUC2
P4
P7
P9
Normal
Primary Tumor
MUC2(Goblet)
High
Low
D
LYZ(Panth)
High
Low
LYZ
(Patient 2)
E
TGFBI(EMT)
High
Low

Fig. S2 Transcriptomic differences between adjacent normal tissues and tumor tissues.

A. tSNE maps of all epithelial cells. Cells were colored with patient and tissue origin. N, normal tissue; PT, primary tumor tissue; LyM, lymph node metastasis; LM, liver metastasis.

B. IHC staining of the enterocyte marker CA2 in adjacent normal tissues and primary tumor tissues of Patients #1 and #3-#6. Scale bar, 100μm.

C. IHC staining of goblet cell marker MUC2 in the adjacent normal tissues and primary tumors of Patient #4, #7 and #9. Scale bar, 100μm. The expression levels of *MUC2* were projected on the tSNE map of epithelial cells. Color from yellow to red represent expression level (log_2(TPM+1)) from low to high.

D. IHC staining of Paneth cell marker LYZ in the adjacent normal tissues, primary tumors and metastatic tumors of Patient #2. Scale bar, 100μm. The expression levels of *LYZ* were projected on the tSNE map of epithelial cells. Color from yellow to red represent expression level (log_2(TPM+1)) from low to high.

E. The expression levels of *TGFBI* were projected on the tSNE map of epithelial cells. Color from yellow to red represent expression level (log_2(TPM+1)) from low to high.

Fig.S3

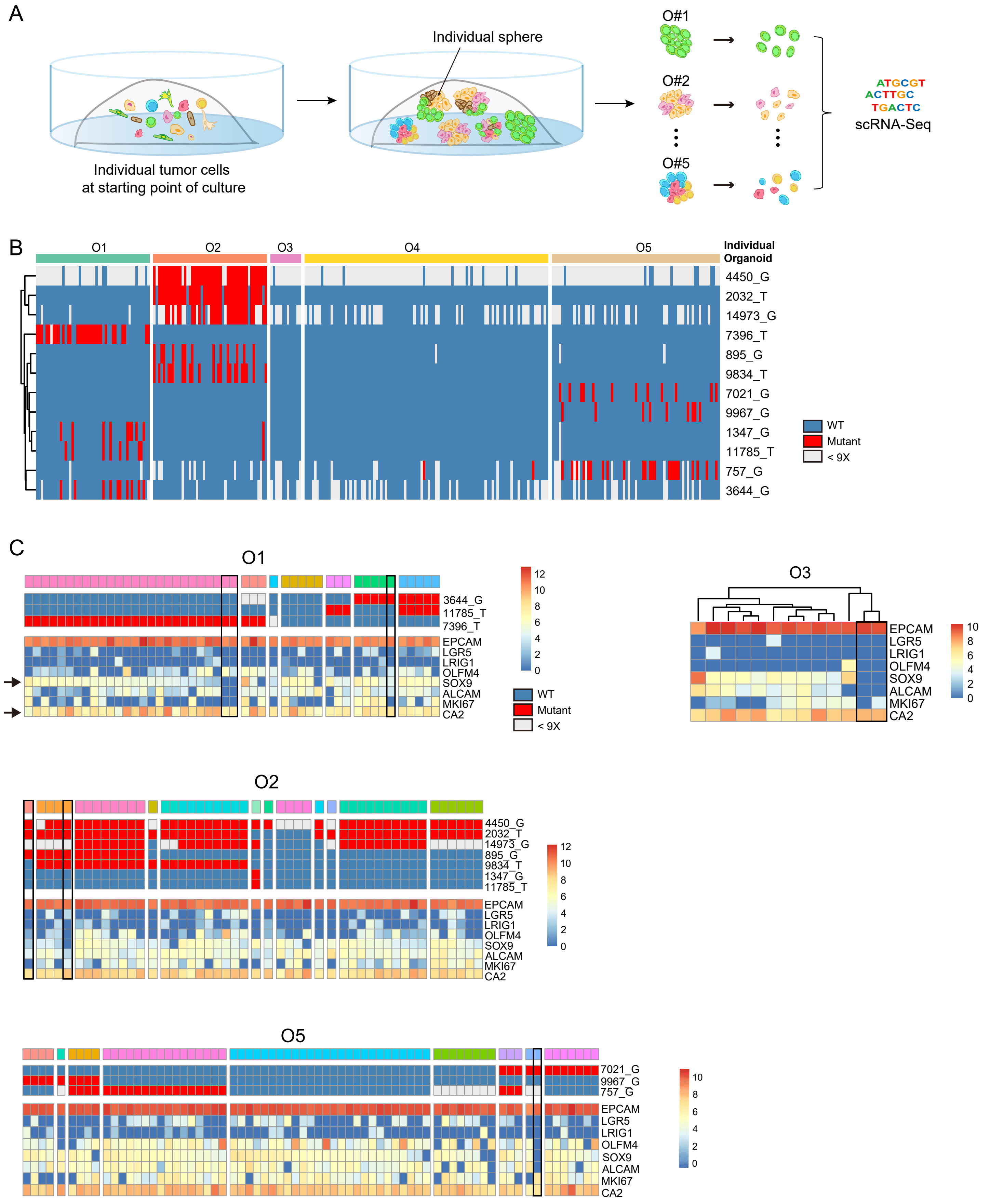
A
Individual tumor cells
at starting point of culture
Individual sphere
O#1
O#2
O#5
ATGCGT
ACTTGC
TGACTC
scRNA-Seq
B
O1
O2
O3
O4
O5
Individual Organoid
4450_G
2032_T
14973_G
7396_T
895_G
9834_T
7021_G
9967_G
1347_G
11785_T
757_G
3644_G
WT
Mutant
< 9X
C
O1
3644_G
11785_T
7396_T
EPCAM
LGR5
LRIG1
OLFM4
SOX9
ALCAM
MKI67
CA2
12
10
8
6
4
2
0
WT
Mutant
< 9X
O3
EPCAM
LGR5
LRIG1
OLFM4
SOX9
ALCAM
MKI67
CA2
10
8
6
4
2
0
O2
4450_G
2032_T
14973_G
895_G
9834_T
1347_G
11785_T
EPCAM
LGR5
LRIG1
OLFM4
SOX9
ALCAM
MKI67
CA2
12
10
8
6
4
2
0
O5
7021_G
9967_G
757_G
EPCAM
LGR5
LRIG1
OLFM4
SOX9
ALCAM
MKI67
CA2
10
8
6
4
2
0

Fig. S3 Lineage tracing and cell-type specific markers' expression patterns of several single organoid spheres.

A. The schematic diagram shows the experimental process of transcriptome sequencing of five single organoid spheres of one patient (O#S5).

B. Heatmap shows the mitochondrial mutations of all cells collected from *in vitro* culture tumor organoids. The bar on the top of the heatmap shows the source of the organoid ball of cells. In total, we performed single-cell transcriptome sequencing on five organoid balls.

C. Heatmap shows mitochondrial mutations and corresponding cell-type specific gene expression in four of five organoids we sequenced. O3 and O4 lack distinguishable mitochondrial mutations, so only heatmaps of gene expression are shown. Color from blue to red represent gene expression level from low to high. As for the mitochondrial mutation heatmap, gray square represents sequenced reads less than 9; blue square represents wild type and red square represent mutation site.

Fig.S4

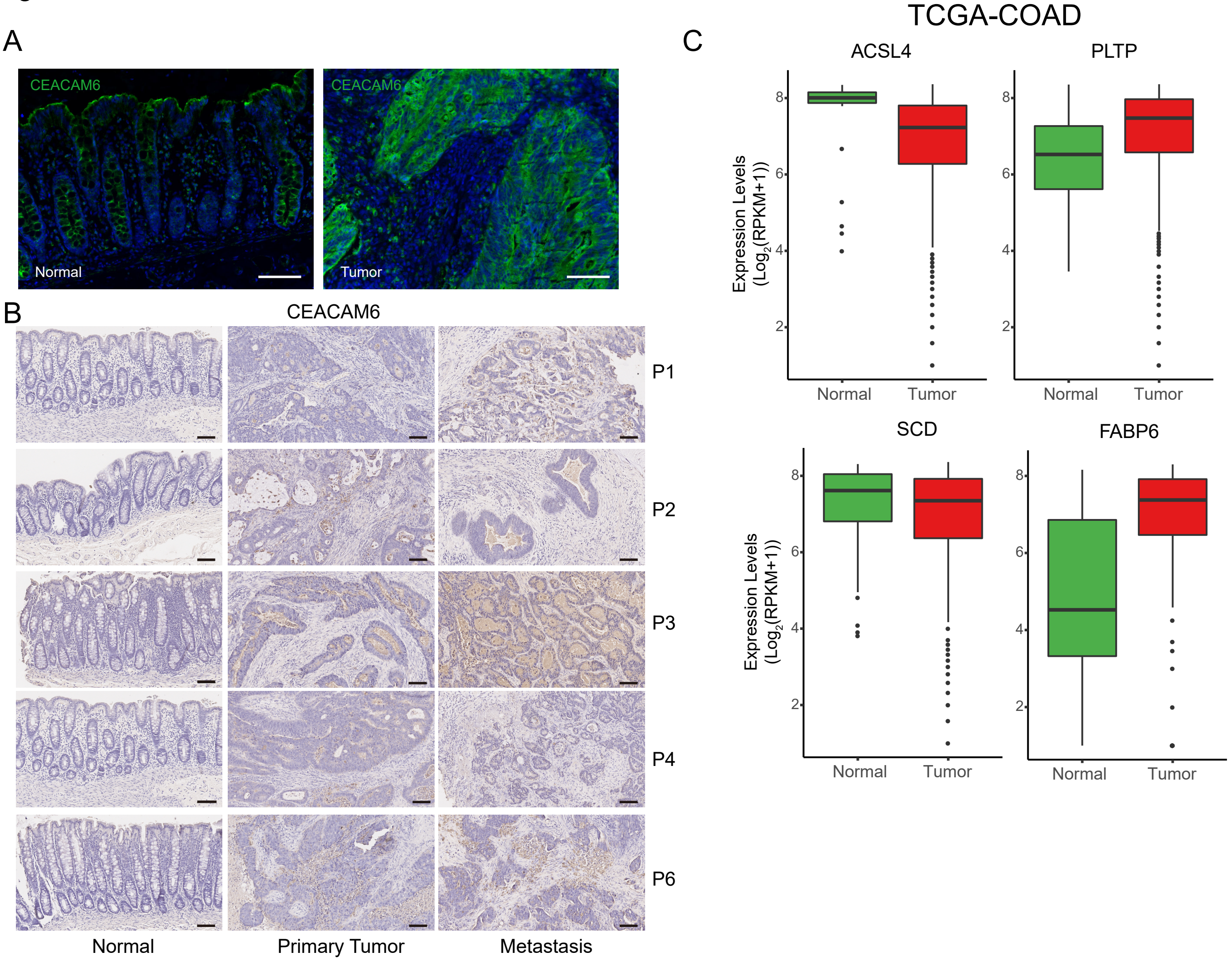
A
CEACAM6
Normal
CEACAM6
Tumor
B
CEACAM6
P1
P2
P3
P4
P6
Normal
Primary Tumor
Metastasis
C
TCGA-COAD
ACSL4
PLTP
SCD
FABP6
Expression Levels
(Log2(RPKM+1))
8
6
4
2
Normal
Tumor

Fig. S4 IHC and immunofluorescence staining verification and survival curve analysis of tumor-specific genes.

A. Immunofluorescence staining of CEACAM6 in adjacent normal tissue and primary tumor tissue for another patient. Scale bar, 100μm.

B. IHC staining of CEACAM6 of adjacent normal tissues, primary tumors and metastatic tumors for Patients # 1-#4 and #6. Scale bar, 100μm.

C. The boxplot shows the expression of PPAR signaling pathway associated genes in TCGA COAD dataset.

Figure S5

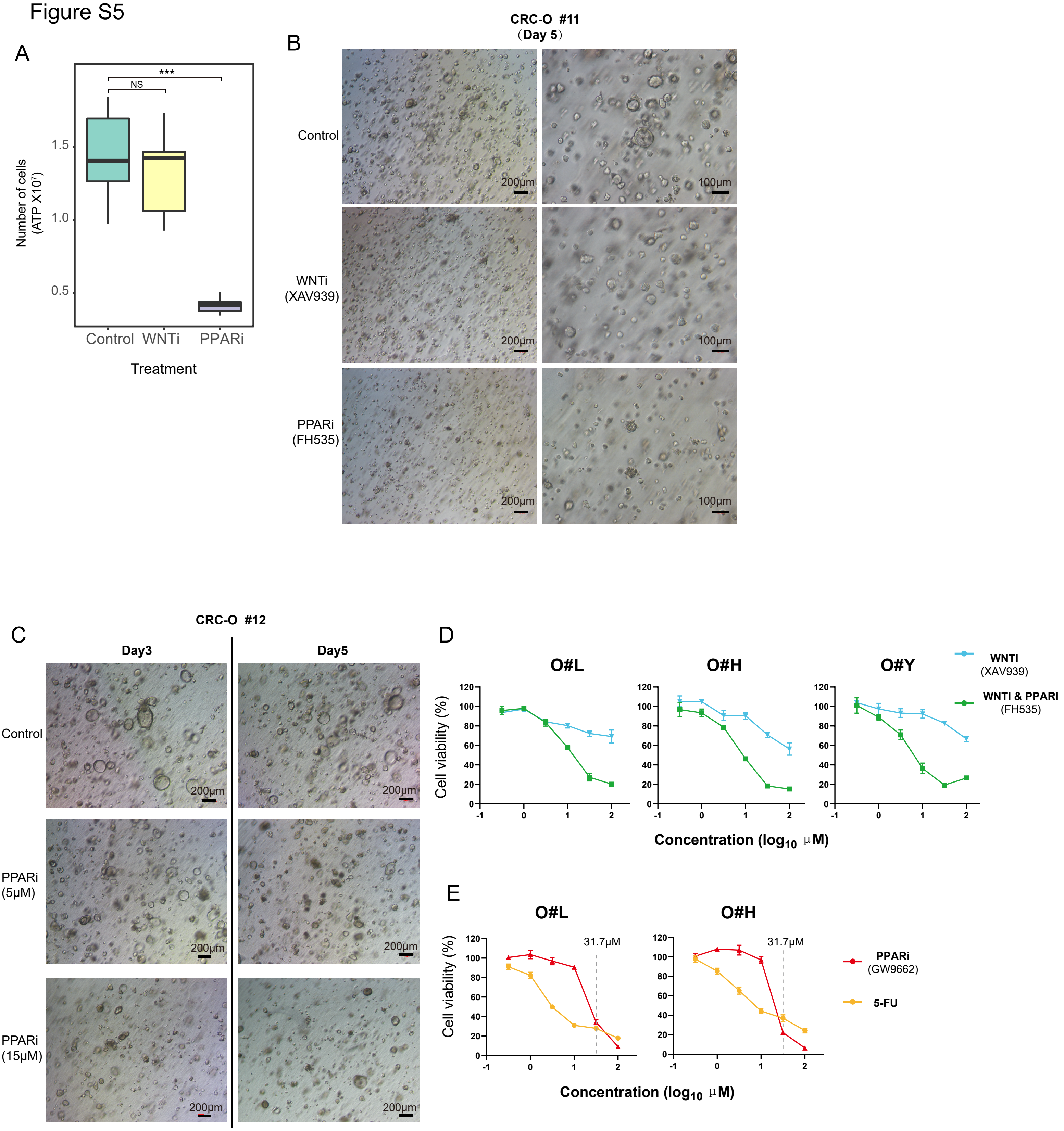

F

KEGG Pathway

	P values
Cell Cycle	1.6 x 10^{-10}
p53 signaling pathway	2.7 x 10^{-4}
PPAR signal pathway	7.4 x 10^{-3}

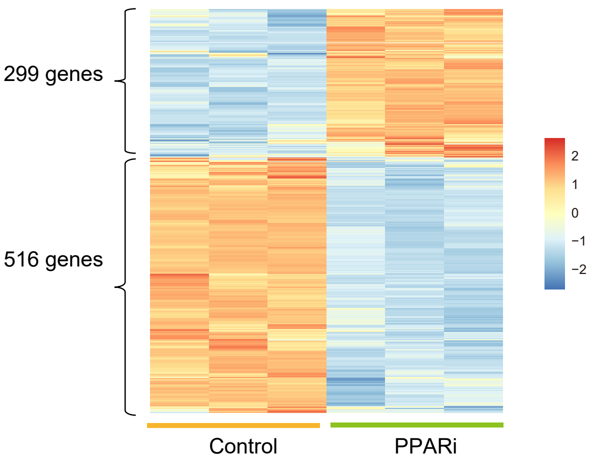

Fig. S5 Comparison with TCGA dataset and inhibition of PPAR signaling pathway on organoid system.

A. Cell viability assay of PPAR signaling pathway inhibitor (FH535)-treated, WNT signaling pathway inhibitor (XAV939)-treated and control patient-derived tumor organoids. The concentration of XAV939 and FH535 were 30 μm and 15 μm respectively. NS represents not significant.

B. Representative bright-field images of PPAR and WNT inhibitor treated and control patient derived tumor organoids. The concentration of XAV939 and FH535 were 30μM and 15μM respectively. *** represent p-value <0.001.

C. Representative bright-field images of different PPAR inhibitor concentrations in treated and control patient derived tumor organoids (scale bars, 200 μm).

D. The dot plot showing the cell viability of tumor organoid that threated by different drugs at different concentrations. XAV939 is an inhibitor of WNT signaling pathway. FH535 is a dual-inhibitor of both WNT and PPAR signaling pathway. The x axis represents the log10 value of each drug concentration. Different colors represent the cell viability after treatment with different drugs. Both O#L and O#H contained *APC* mutations based on the WES result (data not shown). O#H contained p.Gln1378Ter mutation in *APC* gene, while O#L contained p.Arg232Ter and p.Leu1280ValfsTer4 in *APC* gene.

E. The dot plot showing the cell viability of tumor organoid that threated by different drugs at different concentrations. GW9662 is an inhibitor of PPAR signaling pathway. 5-FU is a drug commonly used clinically to treat tumors. The x axis represents the log10 value of each drug concentration. Different colors represent the cell viability after treatment with different drugs.

F. Heatmap showing DEGs between PPAR inhibitor (FH535) treated and control tumor organoids. Colors from orange to blue represent expression levels from low to high.

Fig.S6

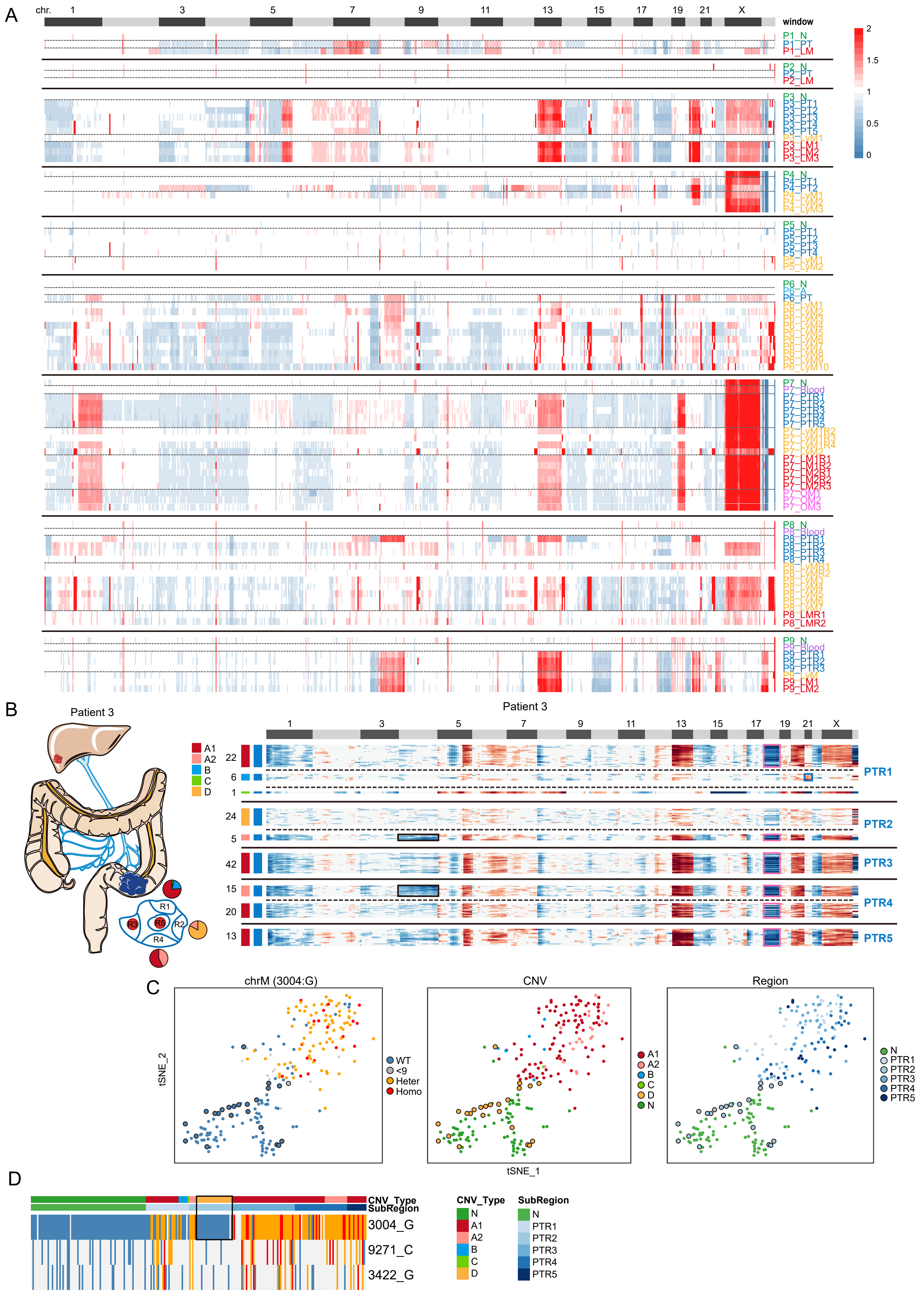

A
chr.
window
P1_N
P1_PT
P1_LM
P2_N
P2_PT
P2_LM
P3_N
P3_PT1
P3_PT2
P3_PT3
P3_PT4
P3_PT5
P3_LyM1
P3_LM1
P3_LM2
P3_LM3
P4_N
P4_PT1
P4_PT2
P4_LyM1
P4_LyM2
P4_LyM3
P5_N
P5_PT1
P5_PT2
P5_PT3
P5_PT4
P5_LyM1
P5_LyM2
P6_N
P6_A
P6_PT
P6_LyM1
P6_LyM10
P7_N
P7_Blood
P7_PTR1
P7_PTR2
P7_PTR3
P7_PTR4
P7_PTR5
P7_LM1R1
P7_LM1R2
P7_LM2R1
P7_LM2R2
P7_LM2R3
P7_OM1
P7_OM2
P7_OM3
P8_N
P8_Blood
P8_PTR1
P8_PTR2
P8_PTR3
P8_PTR4
P8_LyMR1
P8_LyMR2
P8_LMR1
P8_LMR2
P9_N
P9_Blood
P9_PTR1
P9_PTR2
P9_PTR3
P9_LyM
P9_LM1
P9_LM2
2
1.5
1
0.5
0
B
Patient 3
R1
R2
R3
R4
R5
A1
A2
B
C
D
PTR1
PTR2
PTR3
PTR4
PTR5
C
chrM (3004:G)
CNV
Region
tSNE_2
tSNE_1
WT
<9
Heter
Homo
N
D
3004_G
9271_C
3422_G
CNV_Type
SubRegion

Fig. S6 CNV pattern verification by WGS and tumor lineage inferences of Patient #3 by single-cell RNA-Seq data.

A. Heatmap showing the CNV pattern inferred by WGS data. Copy number gain and copy number loss were indicated with red and blue respectively. Different tissues were divided by black dashed lines, and samples collected from different patients were divided by bold black solid lines. The squares above the heat map represent different chromosomes, black squares represent odd-numbered chromosomes and chromosome X, and light gray squares represent even-numbered chromosomes and chromosome Y. N, adjacent normal tissues. PT, primary tumors. LyM, lymph node metastasis. LM, liver metastasis. OM, omentum metastasis.

B. CNV patterns inferred by single-cell RNA data, and sampling strategy of Patient #3. Copy number gain and copy number loss were indicated with red and blue respectively. Different tissues were divided by bold black solid lines, and different clones within the same tissue block were divided by black dashed lines. The squares above the heat map represent different chromosomes, black squares represent odd-numbered chromosomes and chromosome X, and light gray squares represent even-numbered chromosomes and chromosome Y. The squares on the left of the heat map represent different subclones, and the same color represents the same clone. The most obvious copy number differences between different clones were highlighted by different colored boxes.

C. The tSNE map of Patient #3 epithelial cells. Cells were colored by mitochondrial mutation, CNV type and cell origin. The cells from subclone Dare enlarged and highlighted with a black border. D-types cells were collected from primary tumor (PTR2) and exhibited no obvious CNV. In addition, the mitochondrial mutations of D-types cells are also wild-type, and the transcriptome expression is much similar to that of normal cells. This indicates that the mutation status of mitochondria is consistent with the characteristics of the cell's genome and transcriptome.

D. Heatmap showing selected mitochondrial mutations of Patient #3. Blue, orange, red and gray represent wild type, heterozygous, homozygous mutations and undefined (read depth < 9x) respectively. The bar above the heat map shows the CNV subclones and cell origins.

Fig.S7

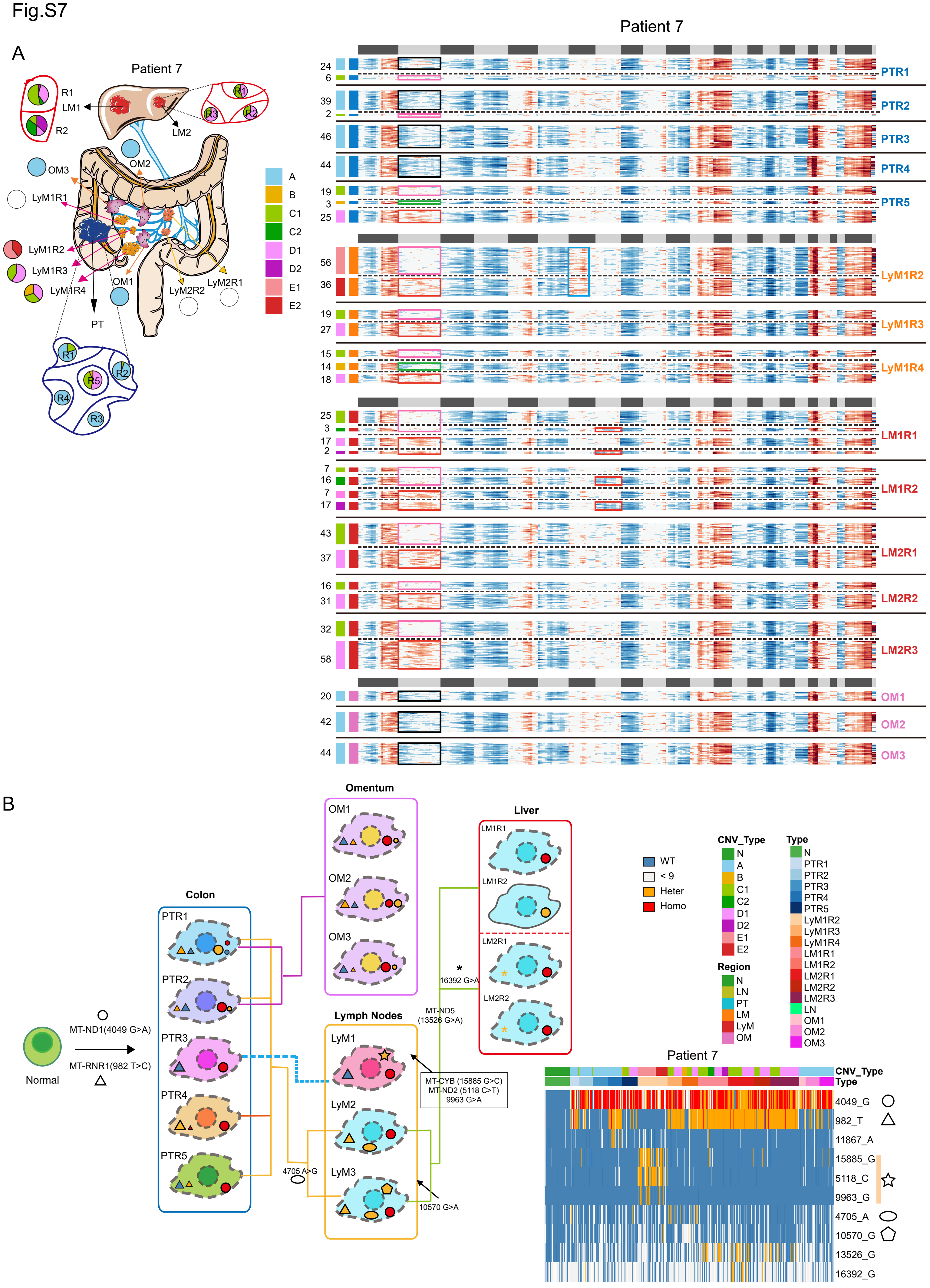

A
Patient 7
R1
LM1
R2
LM2
OM3
OM2
LyM1R1
LyM1R2
LyM1R3
LyM1R4
OM1
LyM2R2
LyM2R1
PT
R1
R2
R3
R4
R5
A
B
C1
C2
D1
D2
E1
E2
PTR1
PTR2
PTR3
PTR4
PTR5
LyM1R2
LyM1R3
LyM1R4
LM1R1
LM1R2
LM2R1
LM2R2
LM2R3
OM1
OM2
OM3
B
Omentum
Liver
Colon
Lymph Nodes
Normal
MT-ND1(4049 G>A)
MT-RNR1(982 T>C)
16392 G>A
MT-ND5 (13526 G>A)
MT-CYB (15885 G>C)
MT-ND2 (5118 C>T)
9963 G>A
4705 A>G
10570 G>A
LyM1
LyM2
LyM3
WT
< 9
Heter
Homo
CNV_Type
Type
Region
N
LN
PT
LM
LyM
OM
4049_G
982_T
11867_A
15885_G
5118_C
9963_G
4705_A
10570_G
13526_G
16392_G

Fig. S7 Tumor lineage inferences of Patient # 7 by mitochondrial mutations and CNVs.

A. CNV patterns and sampling strategy of Patient #7. Pie plot shows the proportion of different subcloned cells in each tumor tissue. Copy number gain and copy number loss were indicated with red and blue respectively. Different tissues were divided by bold black solid lines, and different clones within the same tissue block were divided by black dashed lines. The squares above the heat map represent different chromosomes, black squares represent odd-numbered chromosomes and chromosome X, and light gray squares represent even-numbered chromosomes and chromosome Y. The squares on the left of the heat map represent different subclones, and the same color represents the same clone. The most obvious copy number differences between different clones were highlighted by different colored boxes. PT, primary tumor and PTR1-PTR5 represent different regions of single primary tumor tissues. LyM, lymph node metastasis, and LyM1R2-LyM1R4 represent three different regions of LyM1. LM, liver metastasis, LM1 and LM2 represent two separated liver metastatic tumors, LM1R1-LM1R2 represent two different regions of LM1, and LM2R1-LM2R3 represent three different regions of LM2. OM, omentum metastasis, OM1-OM3 represent three different omentum metastatic tumors.

B. The diagram on the left side showing tumor metastasis path of Patient #7. The heatmap on the right side shows selected mitochondrial mutations in Patient #7. PT, primary tumor. LyM, lymph node metastasis. LM, liver metastasis. OM, omentum metastasis. LN, normal adjacent tissue collected from liver.

Fig.S8

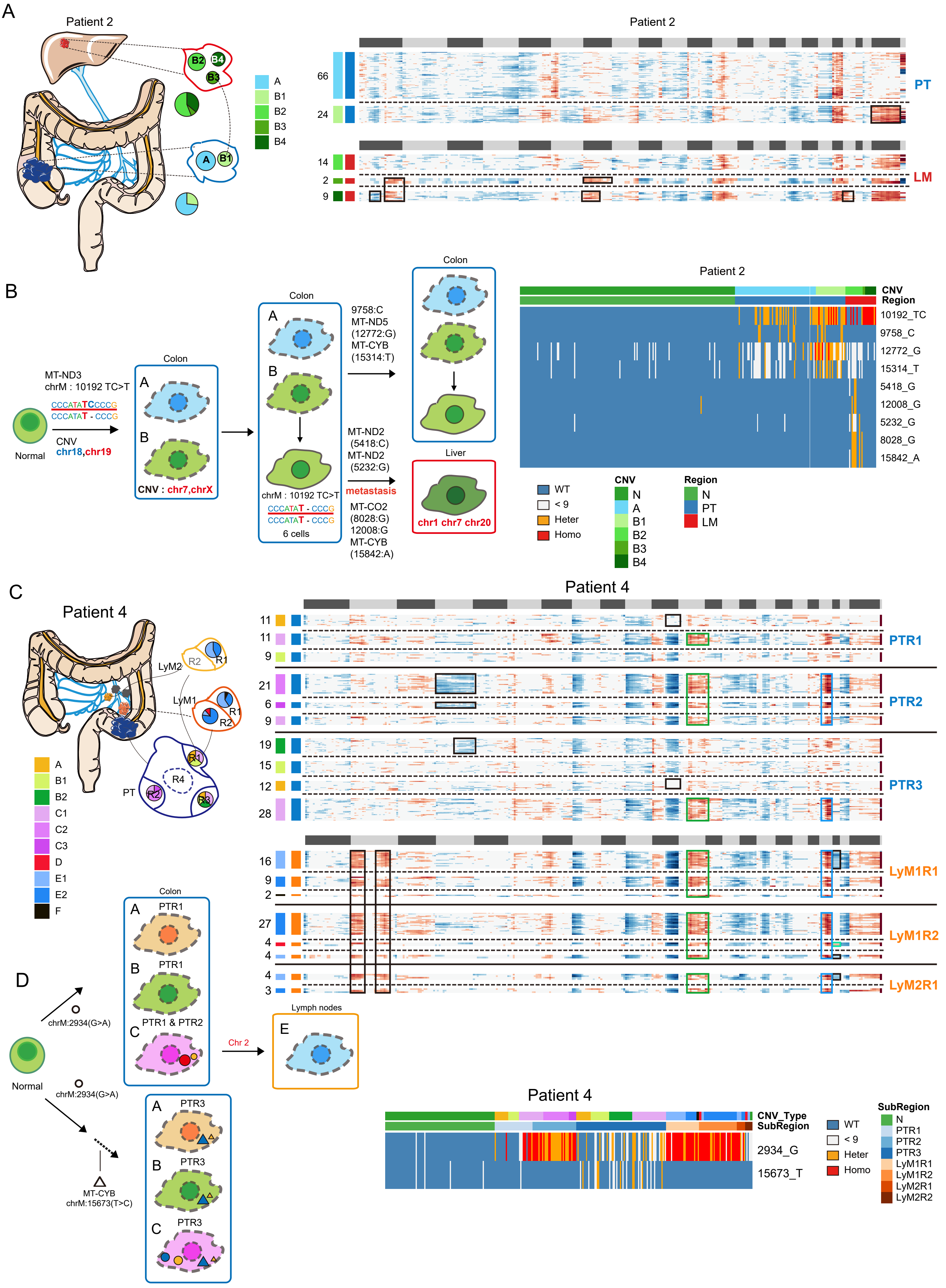
A
Patient 2
A
B1
B2
B3
B4
PT
LM
B
Normal
MT-ND3
chrM : 10192 TC>T
CNV
chr18,chr19
Colon
CNV : chr7,chrX
9758:C
MT-ND5
(12772:G)
MT-CYB
(15314:T)
MT-ND2
(5418:C)
MT-ND2
(5232:G)
metastasis
MT-CO2
(8028:G)
12008:G
MT-CYB
(15842:A)
chrM : 10192 TC>T
6 cells
Liver
chr1 chr7 chr20
CNV
Region
10192_TC
9758_C
12772_G
15314_T
5418_G
12008_G
5232_G
8028_G
15842_A
WT
< 9
Heter
Homo
N
PT
LM
C
Patient 4
LyM2
LyM1
PT
PTR1
PTR2
PTR3
LyM1R1
LyM1R2
LyM2R1
A
B1
B2
C1
C2
C3
D
E1
E2
F
D
Normal
chrM:2934(G>A)
MT-CYB
chrM:15673(T>C)
PTR1 & PTR2
Chr 2
Lymph nodes
CNV_Type
SubRegion
2934_G
15673_T
LyM2R2

Fig. S8 Tumor lineage inferences of Patient #2 and Patient #4 by mitochondrial mutations and CNVs.

A. CNV patterns and sampling strategy of Patient #2. Different subclones with distinct CNV patterns are found in primary tumor and liver metastases. PT: primary tumor. LyM: lymph node metastasis. LM: liver metastasis. The pie charts reflect the proportion of different subclones in each region. Copy number gain and copy number loss were indicated with red and blue respectively. Different tissues were divided by bold black solid lines, and different clones within the same tissue block were divided by black dashed lines. The squares above the heat map represent different chromosomes, black squares represent odd-numbered chromosomes and chromosome X, and light gray squares represent even-numbered chromosomes and chromosome Y. The squares on the left of the heat map represent different subclones, and the same color represents the same clone. The most obvious copy number differences between different clones were highlighted by different colored boxes.

B. The diagram on the left side shows the tumor metastasis path of Patient #2. The heatmap on the right side shows selected mitochondrial mutations in Patient #2. N, adjacent normal tissue; PT, primary tumor; LM, liver metastasis. Major copy number change and distinguishable mitochondrial mutations were showed in the diagram in the left. CNV subclones and cell origins are showed above the heat map.

C. CNV patterns and sampling strategy of Patient #4. Copy number gain and copy number loss were indicated with red and blue respectively. Different tissues were divided by bold black solid lines, and different clones within the same tissue block were divided by black dashed lines. The squares above the heat map represent different chromosomes, black squares represent odd-numbered chromosomes and chromosome X, and light gray squares represent even-numbered chromosomes and chromosome Y. The squares on the left of the heat map represent different subclones, and the same color represents the same clone. The most obvious copy number differences between different clones were highlighted by different colored boxes. PT, primary tumor and PTR1-PTR3 represent different regions of single primary tumor tissues. LyM, lymph node metastasis, LyM1 and LyM2 represent two different visible lymph nodes and LyM1R1 and LyM1R2 represent two regions of LyM1.

D. The diagram on the left side shows the tumor metastasis path of Patient #4. The heatmap on the right side shows selected mitochondrial mutations in Patient #4.

Circle and triangle represent mutation state of 2,934 and 15,673 site respectively and the fill color of these shapes represent wild-type(blue) and heterozygous (orange), homozygous (red) mutation. PT, primary tumor; LyM, lymph node metastasis.

Fig.S9

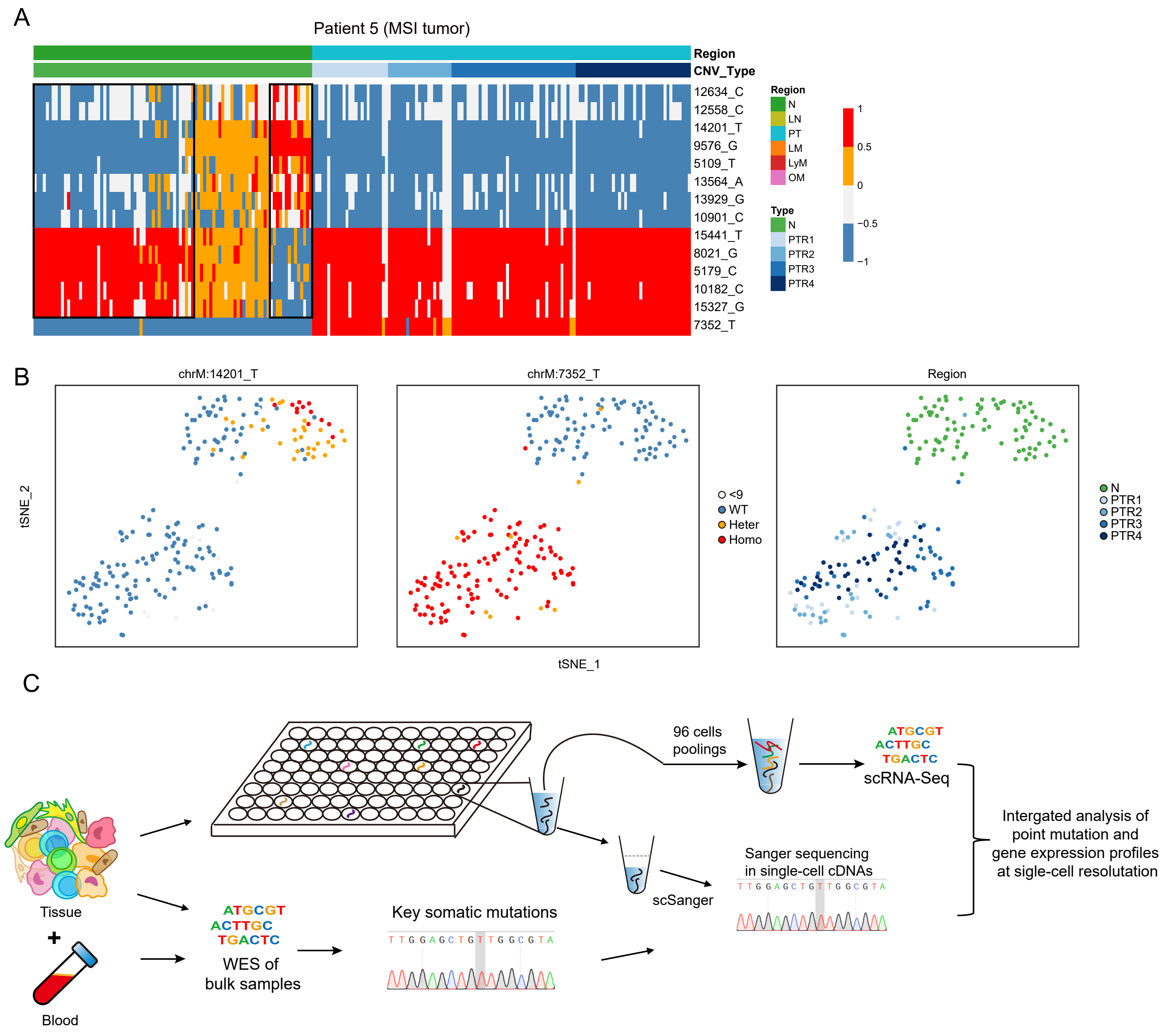
A
Patient 5 (MSI tumor)
Region
CNV_Type
12634_C
12558_C
14201_T
9576_G
5109_T
13564_A
13929_G
10901_C
15441_T
8021_G
5179_C
10182_C
15327_G
7352_T
Region
N
LN
PT
LM
LyM
OM
Type
N
PTR1
PTR2
PTR3
PTR4
1
0.5
0
−0.5
−1
B
chrM:14201_T
chrM:7352_T
Region
tSNE_2
tSNE_1
<9
WT
Heter
Homo
N
PTR1
PTR2
PTR3
PTR4
C
96 cells
poolings
ATGCGT
ACTTGC
TGACTC
scRNA-Seq
Intergated analysis of
point mutation and
gene expression profiles
at sigle-cell resolutation
Sanger sequencing
in single-cell cDNAs
scSanger
Tissue
Blood
WES of
bulk samples
Key somatic mutations

Fig. S9 Normal epithelial cell-specific mitochondrial mutations existed in MSI-H tumor and experimental flowchart for single-cell cDNA Sanger sequencing verification.

A. Heatmap showing the mitochondrial mutation of Patient #5, whose tumor was an MSI tumor. All tumor cells had *MT-CO1* (chrM:7352) mutations. In addition, parts of normal tissue had normal-specific mutations on *MT-ND2* (chrM:5109), *MT-ND5* (chrM:12634, 12558,13564 and 13929) and *MT-ND6* (chrM:14201).

B. The tSNE clustering of Patient #5 epithelial cells. Cells were colored by mitochondrial mutations (chrM: 14201_T and chrM: 7352_T) and cell origin. N, adjacent normal tissue. PT, primary tumor and PTR1-PTR4 represent four different regions of primary tumor.

C. The schematic diagram shows that process of single-cell cDNA sanger verification. The transcriptome of single cells is amplified and barcoded in a single PCR tube. Half of the transcripts in a single tube are pooled with transcripts of other cells to construct the sequence library, and the other half of the transcripts are used for Sanger sequencing verification.

Fig.S10

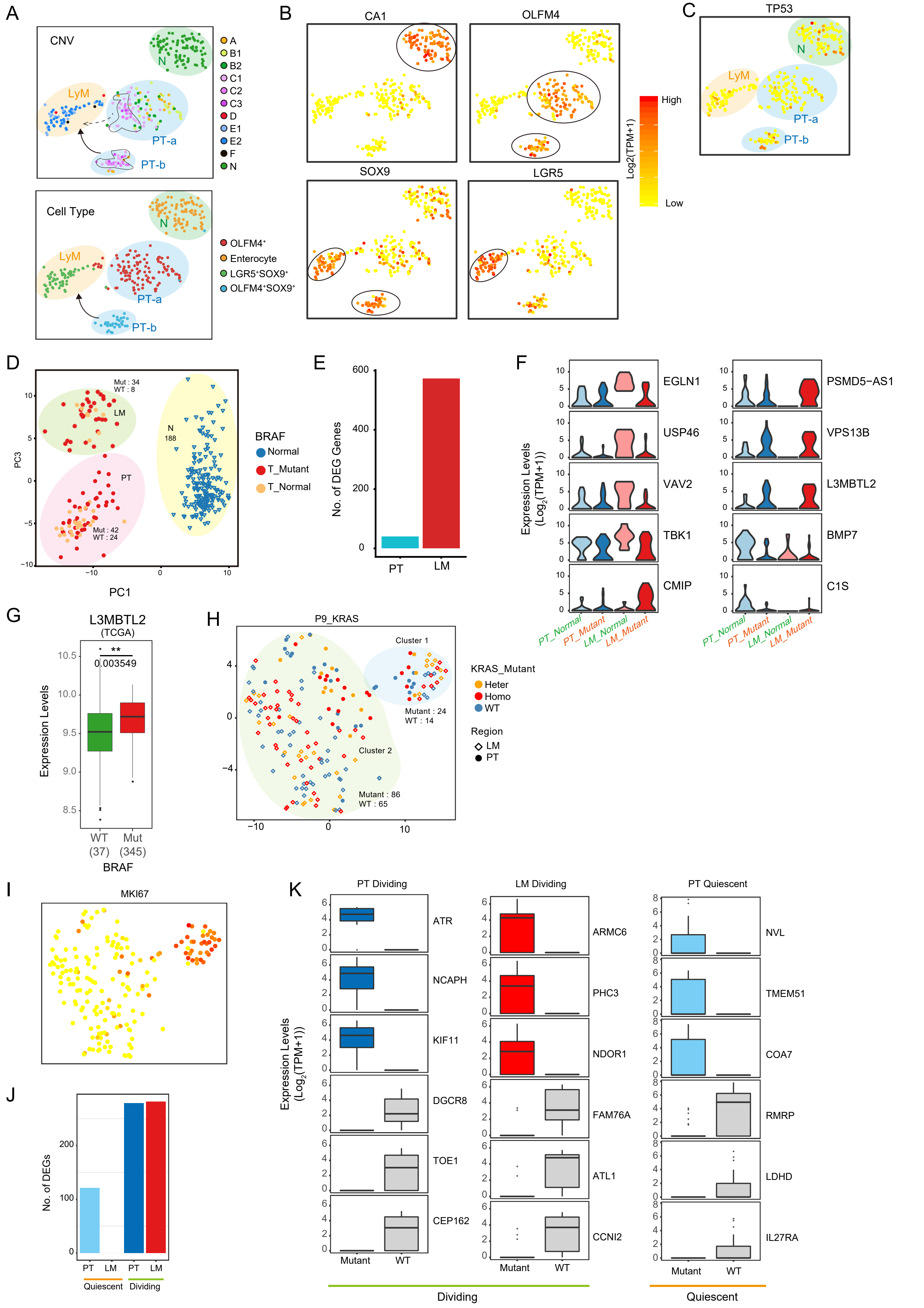
A
CNV
N
LyM
PT-a
PT-b
A
B1
B2
C1
C2
C3
D
E1
E2
F
N
Cell Type
OLFM4+
Enterocyte
LGR5+SOX9+
OLFM4+SOX9+
B
CA1
OLFM4
SOX9
LGR5
High
Low
Log2(TPM+1)
C
TP53
D
Mut : 34
WT : 8
LM
N
188
PT
Mut : 42
WT : 24
PC3
PC1
BRAF
Normal
T_Mutant
T_Normal
E
No. of DEG Genes
PT
LM
F
Expression Levels
(Log2(TPM+1))
EGLN1
USP46
VAV2
TBK1
CMIP
PSMD5-AS1
VPS13B
L3MBTL2
BMP7
C1S
PT_Normal
PT_Mutant
LM_Normal
LM_Mutant
G
L3MBTL2
(TCGA)
**
0.003549
Expression Levels
WT
(37)
Mut
(345)
BRAF
H
P9_KRAS
Cluster 1
Mutant : 24
WT : 14
Cluster 2
Mutant : 86
WT : 65
KRAS_Mutant
Heter
Homo
WT
Region
LM
PT
I
MKI67
J
No. of DEGs
PT
LM
PT
LM
Quiescent
Dividing
K
PT Dividing
LM Dividing
PT Quiescent
Expression Levels
(Log2(TPM+1))
ATR
NCAPH
KIF11
DGCR8
TOE1
CEP162
ARMC6
PHC3
NDOR1
FAM76A
ATL1
CCNI2
NVL
TMEM51
COA7
RMRP
LDHD
IL27RA
Mutant
WT
Dividing
Quiescent

Fig. S10 The relationship between $BRAF^{V600E}$, $KRAS^{G12V}$ mutations and gene expression profiles.

A. The tSNE map of Patient #4, which has a *TP53* frame-shift mutation. Cells were colored according to merge CNV types and cell types.

B. The expression levels of *TP53* was projected on tSNE maps. Colors from yellow to red represent expression levels from low to high.

C. The expression levels of intestinal cell type markers *CA1* (enterocyte cell marker), *OLFM4* (stem/progenitor cell marker), *SOX9* (stem/progenitor cell marker) and *LGR5* (stem cell marker) were projected on tSNE maps. Colors from yellow to red represent expression levels from low to high.

D. PCA clustering of Patient #1 epithelial cells. Cells were colored by *BRAF* mutation and cell origin.

E. Bar plot showing the number of $BRAF^{V600E}$-$BRAF^{WT}$ DGEs identified for primary tumors and liver metastatic tumors.

F. The violin plot showing DEGs among PT $BRAF^{V600E}$, PT $BRAF^{WT}$, LM $BRAF^{V600E}$ and LM $BRAF^{WT}$ epithelial cells. N, adjacent normal tissue. PT, primary tumor. LM, liver metastasis.

G. Box plot shows the expression levels of *L3MBTL2* in BRAF^{600WT} and BRAF^{V600E} in bulk TCGA-COAD dataset.

H. The tSNE clustering of Patient #9 epithelial cells. Cells were colored according to the *KRAS* mutations and the shape of the dot represents cell origin.

I. The expression level of cell proliferate marker *MKI67* was projected to the tSNE map of Patient #9.

J. Bar plot showing the number of $KRAS^{G12V}$-$KRAS^{WT}$ EGs identified for dividing cells and quiescent cells in different tumor regions.

K. The box plot shows DEGs between $KRAS^{G12V}$ and $KRAS^{WT}$ in different cases.
